# Relations of advanced glycation endproducts and dicarbonyls with endothelial dysfunction and low-grade inflammation in individuals with end-stage renal disease in the transition to renal replacement therapy: A cross-sectional observational study

**DOI:** 10.1371/journal.pone.0221058

**Published:** 2019-08-13

**Authors:** Remy J. H. Martens, Natascha J. H. Broers, Bernard Canaud, Maarten H. L. Christiaans, Tom Cornelis, Adelheid Gauly, Marc M. H. Hermans, Constantijn J. A. M. Konings, Frank M. van der Sande, Jean L. J. M. Scheijen, Frank Stifft, Joris J. J. M. Wirtz, Jeroen P. Kooman, Casper G. Schalkwijk

**Affiliations:** 1 Department of Internal Medicine, Division of Nephrology, Maastricht University Medical Center+, the Netherlands; 2 CARIM School for Cardiovascular Diseases, Maastricht University, Maastricht, the Netherlands; 3 NUTRIM School of Nutrition and Translational Research in Metabolism, Maastricht University, Maastricht, the Netherlands; 4 Medical Office EMEA, Fresenius Medical Care Deutschland GmbH, Bad Homburg, Germany; 5 Montpellier University, School of Medicine, Montpellier, France; 6 Department of Nephrology, Jessa Hospital, Hasselt, Belgium; 7 Department of Internal Medicine, Division of Nephrology, Viecuri Medical Center, Venlo, the Netherlands; 8 Department of Internal Medicine, Division of Nephrology, Catharina Hospital Eindhoven, Eindhoven, the Netherlands; 9 Department of Internal Medicine, Maastricht University Medical Center+, Maastricht, the Netherlands; 10 Department of Internal Medicine, Division of Nephrology, Zuyderland Medical Center, Sittard-Geleen, the Netherlands; 11 Department of Internal Medicine, Division of Nephrology, St. Laurentius Hospital, Roermond, the Netherlands; University of Colorado Denver School of Medicine, UNITED STATES

## Abstract

**Background:**

Cardiovascular disease (CVD) related mortality and morbidity are high in end-stage renal disease (ESRD). The pathophysiology of CVD in ESRD may involve non-traditional CVD risk factors, such as accumulation of advanced glycation endproducts (AGEs), dicarbonyls, endothelial dysfunction (ED) and low-grade inflammation (LGI). However, detailed data on the relation of AGEs and dicarbonyls with ED and LGI in ESRD are limited.

**Methods:**

We examined cross-sectional Spearman’s rank correlations of AGEs and dicarbonyls with serum biomarkers of ED and LGI in 43 individuals with chronic kidney disease (CKD) stage 5 not on dialysis (CKD5-ND). Free and protein-bound serum AGEs (*N*^∈^-(carboxymethyl)lysine (CML), *N*^∈^-(carboxyethyl)lysine (CEL), *N*_δ_-(5-hydro-5-methyl-4-imidazolon-2-yl)ornithine (MG-H1)) and serum dicarbonyls (glyoxal, methylglyoxal, 3-deoxyglucosone) were analyzed with tandem mass spectrometry, and tissue AGE accumulation was estimated by skin autofluorescence (SAF). Further, serum biomarkers of ED and LGI included sVCAM-1, sE-selectin, sP-selectin, sThrombomodulin, sICAM-1, sICAM-3, hs-CRP, SAA, IL-6, IL-8 and TNF-α.

**Results:**

After adjustment for age, sex and diabetes status, protein-bound CML was positively correlated with sVCAM-1; free CEL with sVCAM-1 and sThrombomodulin; glyoxal with sThrombomodulin; and methylglyoxal with sVCAM-1 (correlation coefficients ranged from 0.36 *to* 0.44). In addition, free CML was positively correlated with SAA; protein-bound CML with IL-6; *free* CEL with hs-CRP, SAA and IL-6; free MG-H1 with SAA; protein-bound MG-H1 with IL-6; and MGO with hs-CRP and IL-6 (correlation coefficients ranged from 0.33 *to* 0.38). Additional adjustment for eGFR attenuated partial correlations of serum AGEs and serum dicarbonyls with biomarkers of ED and LGI.

**Conclusions:**

In individuals with CKD5-ND, *higher* levels of serum AGEs and serum dicarbonyls were related to biomarkers of ED and LGI after adjustment for age, sex and diabetes mellitus. Correlations were attenuated by eGFR, suggesting that eGFR confounds and/or mediates the relation of serum AGEs and dicarbonyls with ED and LGI.

## Introduction

Cardiovascular disease (CVD) related mortality and morbidity are high in individuals with end-stage renal disease (ESRD) [1,2]. The pathophysiology of CVD in ESRD may involve traditional and non-traditional CVD risk factors [3–5].

Advanced glycation endproducts (AGEs), dicarbonyls, endothelial dysfunction (ED) and low-grade inflammation (LGI) are potential non-traditional CVD risk factors in ESRD [3–7]. AGEs represent a heterogeneous family of modified proteins, which are formed via several metabolic routes, including the nonenzymatic reaction of proteins with reducing sugars and with dicarbonyls. Dicarbonyls are highly reactive intermediates derived from glucose and lipid oxidation, the three most studied being glyoxal (GO), methylglyoxal (MGO), and 3-deoxyglucosone (3-DG) [8,9].

The pathophysiological mechanisms which link AGEs, dicarbonyls, ED and LGI to CVD may be intertwined. Indeed, AGEs may induce ED and LGI through a change in function of intracellular proteins, by activation of cells via binding to cell-surface receptors such as the receptor for AGEs (RAGE), and by altered extracellular matrix-cell interactions [6,8,10]. In addition, dicarbonyls may induce ED and LGI directly or indirectly through enhancement of AGE formation [10–12]. On the other hand, LGI may stimulate the formation of dicarbonyls and, thus, AGEs [13].

Serum levels of free and protein-bound AGEs[14,15], serum dicarbonyls[16], and tissue AGE accumulation as estimated by skin autofluorescence (SAF)[17] are higher in individuals with ESRD as compared with healthy controls. In addition, ESRD has been characterized as a state of ED and LGI[18,19].

However, studies on the relation of serum AGEs and SAF with ED and LGI in individuals with ESRD [20–30] are hampered/limited by the assessment of a limited panel of AGEs (mostly pentosidine and CML) and/or serum biomarkers of ED and LGI, which may not cover the heterogeneous nature of these concepts (*e*.*g*., individual AGEs may differ in biological activity due to differences in binding properties), and the use of techniques which do not distinguish between specific AGEs or between free and protein-bound AGEs. In addition, data on dicarbonyls are lacking. Further, most studies involved individuals on dialysis and dialysis may confound the relation of AGEs and dicarbonyls with ED and LGI in uremia, for example through clearance of low-molecular weight serum biomarkers including free AGEs and dicarbonyls [31] and exposure to glucose and glucose-derived products from peritoneal dialysis fluids [32].

Therefore, we examined the relation of free and protein-bound serum AGEs, serum dicarbonyls and tissue AGE accumulation (estimated by SAF) with an extensive panel of serum biomarkers of ED and LGI in individuals with chronic kidney disease (CKD) stage 5 who were not on dialysis (CKD5-ND).

## Materials and methods

### Study population and design

This cross-sectional study included participants from two separate observational studies, which have been performed in the southern part of the Netherlands and focused on dialysis initiation (hemodialysis (HD) and peritoneal dialysis (PD)) and kidney transplantation, respectively. These studies were conducted between February 2012 and July 2017, and between October 2013 and January 2018), respectively. The methodology of the individual studies has been published previously [33,34]. A complete overview of in- and exclusion criteria of these studies is also provided in S1 Methods.

**Fig 1:**
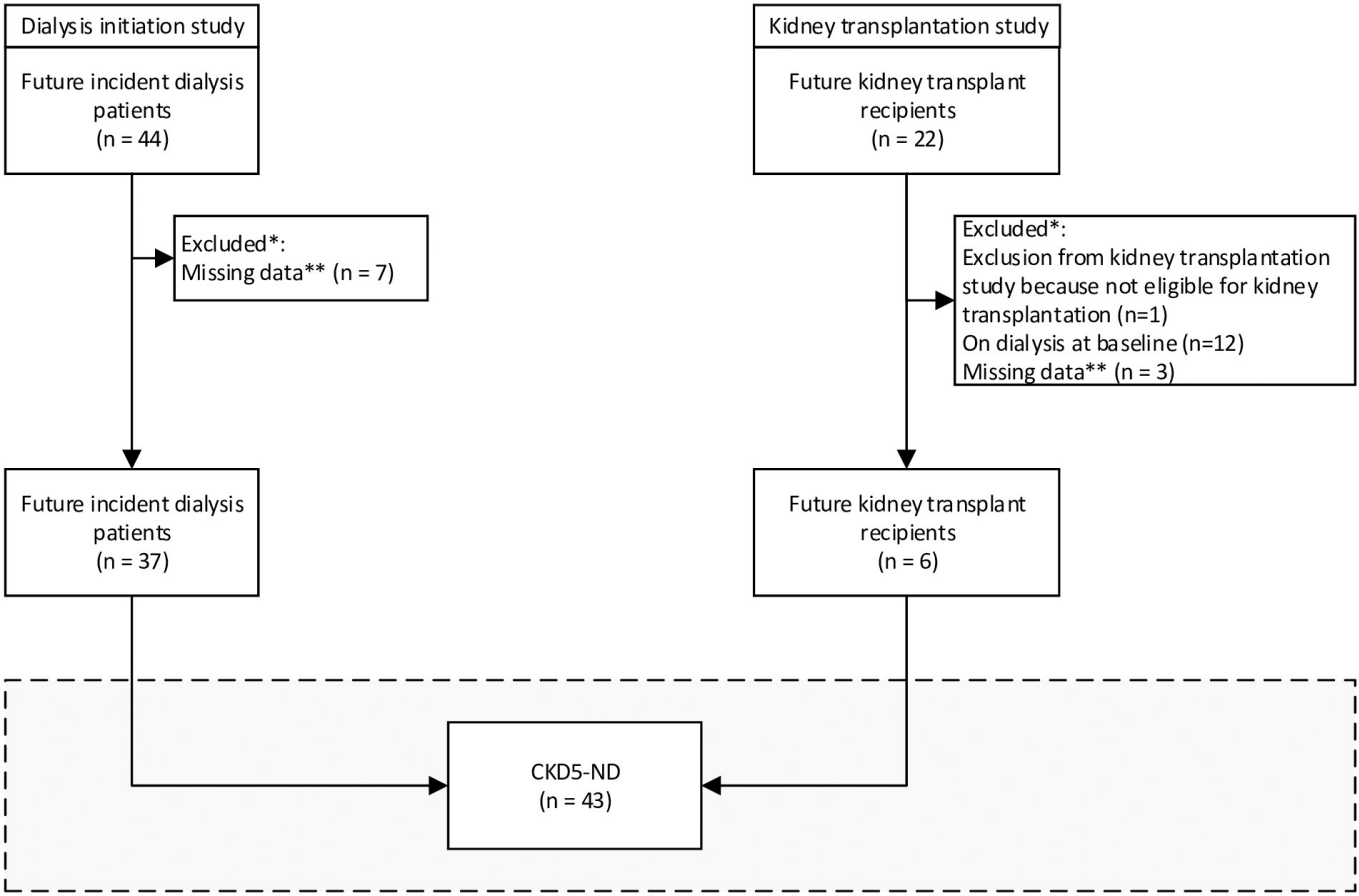
Flowchart depicting the derivation of the study population. Abbreviations: CKD5-ND, chronic kidney disease stage 5 non-dialysis. * Participants were sequentially excluded for the reasons described in the figure (*i*.*e*. counts are mutually exclusive), ** Missing data indicates absence of data on serum biomarkers of endothelial dysfunction and low-grade inflammation or absence of data on serum advanced glycation endproducts, serum dicarbonyls or skin autofluorescence.

Fig 1 is a flowchart showing the derivation of the study population of participants with CKD5-ND. We included baseline data of individuals who would initiate dialysis or receive a kidney transplant. The analyses were performed in the subpopulation with data on serum biomarkers of ED and LGI as well as data on serum AGEs, dicarbonyls or SAF.

Some individuals participated in more than one study (*i*.*e*., incident dialysis patients who subsequently received a kidney transplant or were studied as prevalent dialysis patients). Only data of their first study was analyzed.

Written informed consent was obtained from each patient prior to participation. The studies were approved by the Ethical Committee (NL43381.068.13 and NL33129.068.10) and the Hospital Board of the Maastricht University Medical Center+.

### Study measurements

#### Serum AGEs and dicarbonyls

Serum AGEs and dicarbonyls were measured in stored frozen (-80°C) serum samples.

Serum levels of the free and protein-bound AGEs *N*^∈^-(carboxymethyl)lysine (CML), *N*^∈^-(carboxyethyl)lysine (CEL) and *N*_δ_-(5-hydro-5-methyl-4-imidazolon-2-yl)ornithine (MG-H1), and serum levels of protein-bound lysine were measured with ultraperformance liquid chromatography tandem mass spectrometry. An elaborate description and validation of the method to determine serum levels of the AGEs is provided in S2 Methods. The method for lysine quantification is described in the supplementary material of Hanssen *et al*. [35].

In short, for measurement of protein-bound CML, CEL, MG-H1 and lysine, 25 μl serum was mixed with 50 μl water. After addition of 200 μl sodium borohydride (100 mmol/L) dissolved in borate buffer (pH 9.2, 200 mmol/L), samples were incubated at room temperature for 2 hours. Next, samples were deproteinized with 1000 μL cold (4°C) trifluoroacetic acid. After centrifugation (4300 g, 4°C, 20 min) the supernatant was carefully removed. Samples were then hydrolyzed by adding 500 μL 6 N HCl to the protein pellet and incubated for 20 hours at 90°C. After hydrolysis, 40 μL hydrolysate and 20 μL internal standard was mixed in a reaction vial. For CML, CEL and MG-H1 this mixture was evaporated to dryness under a stream of nitrogen at 70°C and subsequently derivatized with 100 μL 1-butanol:HCl (3:1, v/v) for 90 minutes at 70°C. Samples were then evaporated to dryness under nitrogen and redissolved in 200 μL water. For measurement of lysine, 10 μL hydrolysate was diluted in 800 μL water. Twenty μL of this mixture and 20 μL internal standard was diluted in 500 μL 10 mmol/l ammonia. Derivatized CML, CEL and MG-H1, and underivatized lysine were analyzed by UPLC tandem MS. For measurement of free CML, CEL and MG-H1 50 μl serum was mixed with 25 μL internal standard and subsequently deproteinized with 600 μL of a mixture of methanol and acetonitrile (1:3, by volume) and centrifuged at 14000 rpm for 20 min at room temperature. The supernatant was transferred to a reaction vial and further treated as described for the protein-bound AGEs.

Under these conditions, we found a recovery of 100% for CML and CEL and ~70% for MG-H1. Protein-bound MG-H1 serum concentrations were corrected accordingly. The calibration curves for CML, CEL and MG-H1 were linear over the ranges 5250–0, 6250–0 and 14749–0 nmol/L, respectively. Mean slope (response factor) for protein-bound CML, CEL and MG-H1 as tested in 5 different plasma samples were respectively 1.106 (CV, 4.8%), 1.401 (CV, 6.2%) and 0.780 (CV, 3.3%).

Intra- and inter-assay coefficients of variation were 3.2% and 8.1% for free CML, 3.6% and 8.0% for free CEL, 3.3% and 4.2% for free MG-H1, 2.0% and 5.3% for protein-bound CML, 3.7% and 10.7% for protein-bound CEL, 5.3% and 8.7% for protein-bound MG-H1, and 2.7% and 4.5% for protein-bound lysine, respectively. All protein-bound fractions of AGEs were expressed per mmol lysine to adjust for the amount of protein per sample.[35]

Serum levels of the dicarbonyls glyoxal (GO), methylglyoxal (MGO) and 3-deoxyglucosone (3-DG) were measured with ultraperformance liquid chromatography tandem mass spectrometry as well, as described previously.[36] Intra- and inter-assay coefficients of variation were 4.3% and 14.3% for GO, 2.9% and 7.3% for MGO, and 2.4% and 12.0% for 3-DG, respectively.

#### Skin autofluorescence

AGE accumulation in skin was estimated by SAF with the AGE Reader CU (DiagnOptics Technologies BV, Groningen, the Netherlands; Software version 1.2.0.3, June 2010). The AGE Reader CU is a small desk top unit, which measures SAF non-invasively and thereby assesses skin AGE levels, as described previously.[37] Patients were examined in seated position with the patients’ forearm placed on top of the AGE-reader device.[38] In HD patients, measurements took place on the contralateral side of the shunt arm. In PD patients and healthy controls, there were no restrictions with regard to the measurement arm. SAF was measured three times by the device. The mean SAF of three measurements was used for the statistical analyses.

#### Serum biomarkers of endothelial dysfunction and low-grade inflammation

Soluble vascular cellular adhesion molecule 1 (sVCAM-1), soluble E-selectin (sE-selectin), soluble P-selectin (sP-selectin), soluble Thrombomodulin (sThrombomodulin), soluble intercellular adhesion molecule 1 (sICAM-1), soluble intercellular adhesion molecular 3 (sICAM-3), high-sensitivity C-reactive protein (hs-CRP), serum amyloid A (SAA), interleukin 6 (IL-6), interleukin 8 (IL-8), and tumor necrosis factor alpha (TNF-α) were measured with a single- or multiplex array detection system based on electro-chemiluminescence technology (Meso Scale Discovery SECTOR Imager 2400, Gaithersburg, Maryland, USA). All measurements were performed in duplicate. Inter-assay and intra-assay variations for all the markers were <9%, except the inter-assay variations for sE-selectin (10.1%), sICAM-1 (10.2%), and IL-6 (14.0%).

#### Other laboratory parameters

Serum creatinine was measured with routine laboratory measurements at the time of study participation, except for kidney transplant recipients, for whom these data were retrospectively collected from the health record. GFR was estimated with the CKD-EPI equation based on serum creatinine (eGFR_CKD-EPI_) [39].

#### Clinical characteristics

Origin of renal disease was based on the diagnosis as reported in the patient’s health record and categorized as nephrosclerosis, glomerulosclerosis, hypertensive nephropathy, renovascular disease, diabetic nephropathy, polycystic kidney disease, IgA nephropathy, glomerulonephritis, other, and unknown. History of diabetes mellitus and cardiovascular disease, medication use, history of kidney transplantation, and residual urine output were collected from the patient’s electronic medical record. Body mass index (BMI) was calculated as weight divided by height squared. Fluid overload was measured with the Body Composition Monitor (BCM) (Fresenius Medical Care, Bad Homburg, Germany). Office blood pressure was measured with an electronic sphygmomanometer (Omron M4-I, Omron, Japan).

All participants were requested to be in a fasting state during the measurements. For practical reasons not all patients were in fasting state as requested, for example, individuals with diabetes.

### Statistical analyses

Characteristics of the study population were presented as means with standard deviations (SDs), medians with 25^th^ and 75^th^ percentiles, and numbers with percentages, as appropriate.

First, correlations between of serum AGEs, serum dicarbonyls and SAF with individual serum biomarkers of ED and LGI were evaluated with partial Spearman’s rank correlation coefficients, adjusted for age, sex and diabetes mellitus.

Second, associations of serum AGEs, serum dicarbonyls and SAF with standardized composite scores of ED and LGI were evaluated with linear regression analyses. The use of composite scores may increase statistical efficiency and reduce the influence of biological and analytical variability [40]. For this purpose, levels of each individual biomarker were transformed (inverse transformation for IL-8 and TNF-α, and natural log transformation for the other serum biomarkers of ED and LGI) and standardized. Subsequently, the Z-scores were calculated as the standardized average of the standardized scores of the individual serum biomarkers. The Z-score for ED included sVCAM-1, sE-selectin, sP-selectin, sThrombomodulin, sICAM-1 and sICAM-3, and the Z-score for LGI consisted of hs-CRP, SAA, IL-6, IL-8, TNF-α, sICAM-1 and sICAM-3 [41]. The serum biomarkers sICAM-1 and sICAM-3 were included in the Z-scores for both ED and LGI as both are expressed by endothelial cells and leukocytes [41]. In addition, levels of serum AGEs, serum dicarbonyls and SAF were standardized. For this purpose, data of serum AGEs and dicarbonyls had to be transformed (*e*.*g*., square root transformation for free CML, inverse transformation for protein-bound MG-H1 and 3-DG, and natural log transformation for the other serum AGEs and serum dicarbonyls). For the final analyses, the regression coefficients represent the SD difference in Z-score for ED and LGI per 1 SD higher level of serum AGEs, serum dicarbonyls and SAF. All linear regression analyses were analyzed unadjusted (model 1) and adjusted for age, sex and diabetes mellitus (model 2).

In additional analyses, correlations and associations were adjusted for eGFR_CKD-EPI_ and fluid overload, which may confound and/or mediate the relation of AGEs and dicarbonyls with ED and LGI.

A *P* value < 0.050 was considered statistically significant.

Analyses were conducted in R Version 3.5.1 [42] with RStudio Version 1.1.456 [43] combined with the packages tidyverse, haven, mass, psych and corrplot.

## Results

### Population characteristics

Forty-three participants with CKD5-ND were included in the analyses, of whom 37 were scheduled to initiate dialysis and 6 to pre-emptively receive a kidney transplant. Table 1 shows further characteristics of the entire study population and stratified according to tertiles of eGFR. In general, participants with lower eGFR had higher levels of serum AGEs, serum dicarbonyls, and biomarkers of LGI, as described previously for this study population (submitted for publication), and shown in S1 and S2 Tables.

**Table 1:** Population characteristics.

		Tertiles of eGFR		
	Study population	T1 (highest)	T2 (middle)	T3 (lowest)
	(n = 43)	(n = 14)	(n = 14)	(n = 14)
*Clinical characteristics*				
Age (years)	58.8 ±13.9	55.4 ±16.9	65.1 ±10.7	57.3 ±11.8
Men	29 (67.4%)	6 (42.9%)	12 (85.7%)	11 (78.6%)
Origin of end-stage renal disease:				
Nephrosclerosis	6 (14.0%)	1 (7.1%)	4 (28.6%)	1 (7.1%)
Glomerulosclerosis	2 (4.7%)	1 (7.1%)	0 (0.0%)	2 (14.3%)
Hypertensive nephropathy	2 (4.7%)	1 (7.1%)	0 (0.0%)	1 (7.1%)
Renovascular disease	0 (0.0%)	0 (0.0%)	0 (0.0%)	0 (0.0%)
Diabetic nephropathy	2 (4.7%)	2 (14.3%)	0 (0.0%)	0 (0.0%)
Polycystic kidney disease	11 (25.6%)	2 (14.3%)	4 (28.6%)	5 (35.7%)
IgA nephropathy	2 (4.7%)	1 (7.1%)	1 (7.1%)	0 (0.0%)
Glomerulonephritis	4 (9.3%)	1 (7.1%)	2 (14.3%)	1 (7.1%)
Nephrotic syndrome	6 (14.0%)	3 (21.4%)	2 (14.3%)	0 (0.0%)
Other	1 (2.3%)	0 (0.0%)	1 (7.1%)	0 (0.0%)
Unknown	7 (16.3%)	3 (21.4%)	0 (0.0%)	4 (28.6%)
History of KTx	8 (18.6%)	1 (7.1%)	4 (28.6%)	3 (21.4%)
First future treatment modality				
HD	19 (44.1%)	5 (35.7%)	7 (50.0%)	7 (50.0%)
PD	18 (41.9%)	4 (28.6%)	7 (50.0%)	6 (42.9%)
Preemptive KTx	6 (14.0%)	5 (35.7%)	0 (0.0%)	1 (7.1%)
Non-preemptive KTx	0 (0.0%)	0 (0.0%)	0 (0.0%)	0 (0.0%)
eGFR_CKD-EPI_ (mL/min/1.73m^2^)*	8.6 ±3.1	12.2 ±2.2	8.2 ±0.7	5.6 ±1.1
Residual urine output*	38 (100%)	14 (100%)	12 (100%)	12 (100%)
Residual urine output (mL/24h)*	2000 [1700–2296]	2050 [1688–2247]	1948 [1700–2317]	2250 [1500–2400]
Diabetes mellitus	5 (11.6%)	1 (7.1%)	2 (14.3%)	2 (14.3%)
Cardiovascular disease	14 (32.6%)	4 (28.6%)	6 (42.9%)	4 (28.6%)
BMI (kg/m^2^)	24.8 ±3.9	23.9 ±4.4	24.9 ±3.1	25.2 ±4.2
Fluid overload (L)*	1.3 ±2.0	1.1 ±1.2	1.1 ±2.3	1.8 ±2.5
SBP (mmHg)	147.1 ±23.2	148.1 ±25.8	139.7 ±23.1	151.0 ±19.3
DBP (mmHg)	83.0 ±13.1	82.7 ±11.3	77.7 ±11.7	85.5 ±11.0
RAAS inhibitor use	18 (41.9%)	8 (57.1%)	7 (50.0%)	3 (21.4%)
Statin use	22 (51.2%)	8 (57.1%)	9 (64.3%)	5 (35.7%)
*Advanced glycation endproducts and dicarbonyls*				
CML_free_ (nmol/L)*	1142.2 [765.1–1538.3]	684.7 [489.3–1040.6]	1291.9 [857.6–1588.9]	1511.2 [1168.0–1620.0]
CML_protein-bound_ (nmol/mmol lysine)*	197.3 [163.1–256.6]	166.3 [140.1–194.7]	193.3 [168.1–267.7]	260.5 [202.2–343.5]
CEL_free_ (nmol/L)*	734.7 [520.2–939.4]	499.0 [318.3–693.6]	765.1 [499.8–973.1]	949.8 [779.9–1075.4]
CEL_protein-bound_ (nmol/mmol lysine)*	58.0 [41.8–74.3]	51.5 [36.7–68.9]	50.1 [40.6–59.1]	74.9 [59.4–92.1]
MG-H1_free_ (nmol/L)*	2244.4 [1670.2–3154.6]	1631.4 [1091.2–2222.0]	2310.0 [2028.9–2808.1]	3251.7 [2209.8–4891.9]
MG-H1_protein-bound_ (nmol/mmol lysine)*	59.1 [47.6–69.9]	51.1 [44.6–60.7]	49.7 [46.9–72.9]	66.8 [61.5–87.7]
GO (nmol/L)*	1724.3 [1319.9–2157.4]	1234.4 [1066.4–1680.9]	1799.5 [1626.0–2455.7]	2041.4 [1749.3–2323.2]
MGO (nmol/L)*	1082.0 [856.6–1417.4]	906.2 [681.2–1131.9]	980.1 [826.3–1418.8]	1190.0 [1072.5–1615.3]
3-DG (nmol/L)*	1635.8 [1371.0–1948.5]	1432.4 [1233.8–1970.8]	1635.8 [1531.5–2060.2]	1641.5 [1391.6–2145.5]
SAF (AU)*	3.2 ±0.7	2.8 ±0.6	3.5 ±0.6	3.2 ±0.6
*Serum biomarkers of endothelial dysfunction and low-grade inflammation*				
sVCAM-1 (μg/L)	887.0 [690.0–1063.0]	774.0 [633.8–893.8]	875.5 [732.8–1234.8]	929.0 [820.5–1072.0]
sE-selectin (μg/L)	11.9 [7.8–16.3]	11.0 [6.8–14.1]	14.3 [10.7–16.4]	9.3 [6.8–17.7]
sP-selectin (μg/L)	51.6 [37.2–63.0]	59.1 [40.4–73.8]	52.8 [44.2–59.7]	45.9 [33.1–61.6]
SThrombomodulin (μg/L)	11.7 [9.5–13.8]	10.1 [9.1–12.6]	11.8 [10.6–13.9]	12.4 [10.2–14.4]
sICAM-1 (μg/L)	415.0 [373.0–504.0]	414.0 [375.4–509.3]	420.0 [388.5–525.3]	391.0 [334.0–476.5]
sICAM-3 (μg/L)	1.0 [0.8–1.3]	1.0 [0.9–1.3]	1.0 [0.8–1.3]	1.0 [0.8–1.3]
hs-CRP (mg/L)	3.2 [1.2–7.6]	1.6 [0.7–5.9]	2.6 [1.5–9.6]	4.8 [2.3–13.9]
SAA (mg/L)	6.8 [2.7–14.0]	3.3 [2.1–9.9]	8.2 [2.6–13.3]	11.3 [5.5–43.9]
IL-6 (ng/L)	1.6 [0.7–2.6]	0.8 [0.6–2.1]	1.9 [1.1–2.8]	1.8 [1.1–3.9]
IL-8 (ng/L)	13.8 [11.4–18.4]	12.7 [11.4–17.6]	15.8 [13.3–21.4]	12.5 [10.1–19.0]
TNF-α (ng/L)	5.0 [4.2–6.0]	4.7 [4.0–6.2]	5.1 [4.5–5.5]	4.7 [4.1–6.2]

Data are presented as n (%), mean ± standard deviation, or median [25^th^ percentile– 75^th^ percentile].

Abbreviations: 3-DG, 3-deoxyglucosone; AU, arbitrary units; BMI, body mass index; CEL, *N*^∈^(carboxyethyl)lysine; CML, *N*^∈^(carboxymethyl)lysine; DBP, diastolic blood pressure; eGFR_CKD-EPI_, estimated glomerular filtration rate based on the creatinine CKD-EPI equation; GO, glyoxal; HD, hemodialysis; hs-CRP, high-sensitivity C-reactive protein; IL-6, interleukin 6; IL-8, interleukin 8; KTx, kidney transplantation; MG-H1, *N*_δ_(5-hydro-5-methyl-4-imidazolon-2-yl)ornithine; MGO, methylglyoxal; NA, not applicable; PD, peritoneal dialysis; RAAS, renin angiotensin aldosterone system; SAA, serum amyloid A; SAF, skin autofluorescence; SBP, systolic blood pressure; sE-selectin, soluble E-selectin; sICAM-1, soluble intercellular adhesion molecule 1; sICAM-3, soluble intercellular adhesion molecule 3; sP-selectin, soluble P-selectin; sThrombomodulin, soluble Thrombomodulin; sVCAM-1, soluble vascular cell adhesion molecule 1; TNF-α, tumor necrosis factor alpha.

* Available in n = 42 for eGFR_CKD-EPI_, n = 38 for residual urine output (dichotomous), n = 27 for residual urine output (continuous), n = 42 for fluid overload, n = 42 for plasma AGEs, n = 38 for dicarbonyls, and n = 38 for skin autofluorescence.

### Correlations of advanced glycation endproducts and dicarbonyls with individual serum biomarkers of endothelial dysfunction

**Fig 2:**
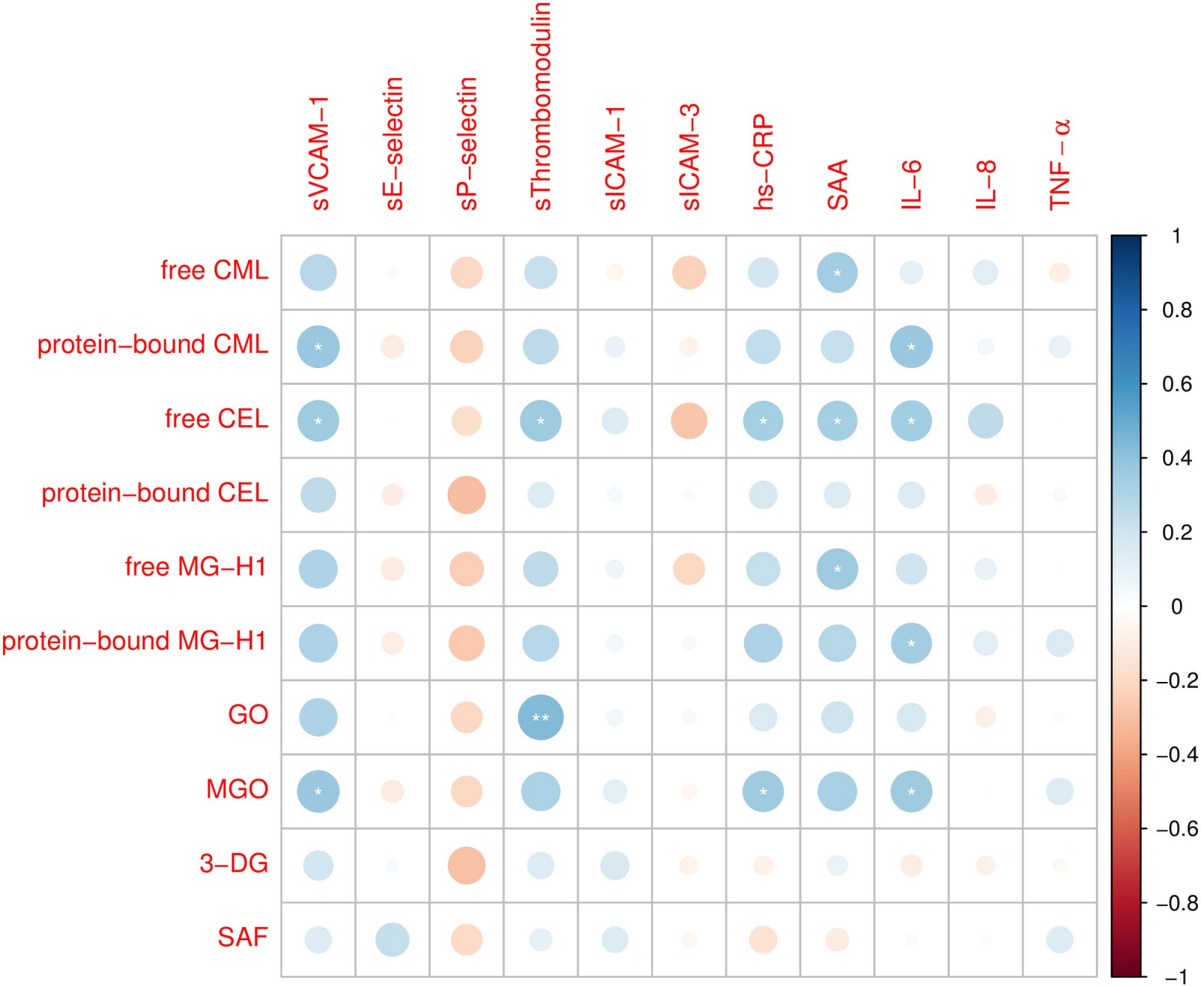
Partial Spearman’s rank correlations of advanced glycation endproducts and dicarbonyls with individual serum biomarkers of endothelial dysfunction and low-grade inflammation. Correlations are adjusted for age, sex and diabetes mellitus. Circle area and color indicate strength of Spearman’s rank correlation coefficients. Analyses are based on n = 42 for advanced glycation endproducts, n = 38 for serum dicarbonyls and n = 38 for skin autofluorescence. Abbreviations: 3-DG, 3-deoxyglucosone; CEL, *N*^∈^(carboxyethyl)lysine; CML, *N*^∈^(carboxymethyl)lysine; GO, glyoxal; hs-CRP, high-sensitivity C-reactive protein; IL-6, interleukin 6; IL-8, interleukin 8; MG-H1, *N*_δ_(5-hydro-5-methyl-4-imidazolon-2-yl)ornithine; MGO, methylglyoxal; SAA, serum amyloid A; SAF, skin autofluorescence; sE-selectin, soluble E-selectin; sICAM-1, soluble intercellular adhesion molecule 1; sICAM-3, soluble intercellular adhesion molecule 3; sP-selectin, soluble P-selectin; sThrombomodulin, soluble Thrombomodulin; sVCAM-1, soluble vascular cell adhesion molecule 1; TNF-α, tumor necrosis factor alpha. * *P* < 0.050, ** *P* < 0.010.

Fig 2 shows partial Spearman’s rank correlations of serum AGEs, serum dicarbonyls and SAF with individual serum biomarkers of ED, adjusted for age, sex and diabetes. Protein-bound CML was positively correlated with sVCAM-1; free CEL with sVCAM-1 and sThrombomodulin; GO with sThrombomodulin; and MGO with sVCAM-1. The corresponding correlation coefficients ranged from 0.36 (*e*.*g*., free CEL with sVCAM-1 and sThrombomodulin) *to* 0.44 (*e*.*g*., GO with sThrombomodulin). Most other measured serum AGEs and serum dicarbonyls were positively correlated with sVCAM-1 and sThrombomodulin as well (Spearman’s ρ 0.15 *to* 0.32), albeit not statistically significantly. In addition, most serum AGEs and serum dicarbonyls showed non-statistically significantly negative correlations with sP-selectin (Spearman’s ρ -0.30 *to* -0.18). Further, SAF was not statistically significantly correlated with the measured serum biomarkers of ED.

### Correlations of advanced glycation endproducts and dicarbonyls with individual serum biomarkers of low-grade inflammation

Fig 2 shows partial Spearman’s rank correlations of serum AGEs, serum dicarbonyls and SAF with individual serum biomarkers of LGI, adjusted for age, sex and diabetes. Free CML was positively correlated with SAA; protein-bound CML with IL-6; free CEL with hs-CRP, SAA and IL-6; free MG-H1 with SAA; protein-bound MG-H1 with IL-6; and MGO with hs-CRP and IL-6. The corresponding correlation coefficients ranged from 0.33 (*e*.*g*., free CEL with hs-CRP and SAA) *to* 0.38 (*e*.*g*., protein-bound CML with IL-6). Other correlations of serum AGEs and serum dicarbonyls with hs-CRP, SAA and IL-6 were not statistically significant, although some were of similar magnitude (*e*.*g*., correlations of protein-bound MG-H1 with hs-CRP and SAA, and of MGO with SAA). Further, SAF was not statistically significantly correlated with the measured serum biomarkers of LGI.

### Associations of advanced glycation endproducts and dicarbonyls with a composite score of endothelial dysfunction

In unadjusted analyses (Table 2, column 3, model 1) and after adjustment for age, sex and diabetes (Table 2, column 3, model 2), none of the measured serum AGEs, serum dicarbonyls or SAF was associated with the composite score of ED.

**Table 2:** Associations of advanced glycation endproducts and dicarbonyls with composite scores of endothelial dysfunction and low-grade inflammation.

		Endothelial dysfunction		Low-grade inflammation	
Biomarker*	Model	Beta (95%CI)	*P* value	Beta (95%CI)	*P* value
CML_free_	1	0.00 (-0.32; 0.33)	0.981	0.09 (-0.23; 0.40)	0.582
	2	0.01 (-0.33; 0.35)	0.955	0.07 (-0.26; 0.40)	0.655
CML_protein-bound_	1	0.11 (-0.21; 0.43)	0.482	0.28 (-0.03; 0.58)	0.071
	2	0.08 (-0.26; 0.43)	0.626	0.24 (-0.09; 0.56)	0.148
CEL_free_	1	0.12 (-0.20; 0.44)	0.441	0.25 (-0.05; 0.56)	0.099
	2	0.14 (-0.21; 0.50)	0.412	0.25 (-0.08; 0.58)	0.139
CEL_protein-bound_	1	0.03 (-0.29; 0.35)	0.851	0.08 (-0.24; 0.39)	0.621
	2	0.06 (-0.28; 0.40)	0.702	0.09 (-0.24; 0.41)	0.596
MG-H1_free_	1	-0.05 (-0.37; 0.27)	0.760	0.12 (-0.19; 0.44)	0.432
	2	-0.04 (-0.38; 0.30)	0.828	0.11 (-0.22; 0.43)	0.514
MG-H1_protein-bound_	1	0.09 (-0.24; 0.41)	0.591	0.32 (0.02; 0.61)	0.039
	2	0.08 (-0.24; 0.41)	0.627	0.30 (-0.01; 0.60)	0.057
GO	1	0.21 (-0.13; 0.56)	0.213	0.15 (-0.19; 0.49)	0.386
	2	0.19 (-0.22; 0.59)	0.352	0.08 (-0.31; 0.47)	0.692
MGO	1	0.18 (-0.17; 0.53)	0.298	0.29 (-0.04; 0.62)	0.086
	2	0.15 (-0.23; 0.54)	0.421	0.30 (-0.06; 0.66)	0.095
3-DG	1	0.16 (-0.19; 0.50)	0.366	-0.06 (-0.40; 0.29)	0.736
	2	0.07 (-0.39; 0.43)	0.760	-0.30 (-0.74; 0.13)	0.162
SAF	1	0.25 (-0.07; 0.56)	0.124	0.20 (-0.13; 0.53)	0.229
	2	0.22 (-0.17; 0.61)	0.257	0.06 (-0.34; 0.47)	0.753

Betas represent the standardized difference in Z scores of endothelial dysfunction and low-grade inflammation per 1 standard deviation higher transformed levels of serum advanced glycation endproducts and serum dicarbonyls, and per 1 standard deviation higher skin autofluorescence. All serum advanced glycation endproducts and serum dicarbonyls were natural log transformed, except for free *N*^∈^(carboxymethyl)lysine (square root transformation), protein-bound MG-H1 (inverse transformation) and 3-deoxyglucosone (inverse transformation). In addition, skin autofluorescence was analyzed on its original scale.

Model 1: unadjusted analyses, model 2: adjusted for age, sex and diabetes mellitus.

Abbreviations: 3-DG, 3-deoxyglucosone; CEL, *N*^∈^(carboxyethyl)lysine; CML, *N*^∈^(carboxymethyl)lysine; GO, glyoxal; MG-H1, *N*_δ_(5-hydro-5-methyl-4-imidazolon-2-yl)ornithine; MGO, methylglyoxal; NA, not applicable; SAF, skin autofluorescence.

* Analyses based on n = 42 for serum AGEs, n = 38 for serum dicarbonyls and n = 38 for skin autofluorescence.

### Associations of advanced glycation endproducts and dicarbonyls with a composite score of low-grade inflammation

In unadjusted analyses (Table 2, column 5, model 1), *higher* protein-bound MG-H1 was associated with a *higher* composite score of LGI. In addition, *higher* protein-bound CML, free CEL, and MGO were associated with a *higher* composite score of LGI, albeit not statistically significantly. Results were largely similar after adjustment for age, sex and diabetes mellitus, although confidence intervals of the regression coefficients became wider (Table 2, column 5, model 2).

### Additional analyses

First, residual plots identified one participant with standardized residuals < -2 for all associations on ED (no evident reason), one with standardized residuals > 2 for all associations on ED and LGI (recent percutaneous transluminal angioplasty of the dialysis shunt and history of recurring infected liver cysts; most recent episode six months before study participation) and one with standardized residuals > 2 for all associations on LGI, except for that of MGO (recent viral infection). S3 Table shows further characteristics of the outliers. After exclusion of outliers (S4 Table), most associations of serum AGEs and serum dicarbonyls with the composite scores of ED and LGI became somewhat stronger. In addition, the associations of GO with the composite score of ED, and the associations of protein-bound CML, free CEL, protein-bound MG-H1, GO and MGO with the composite score of LGI became (borderline) statistically significant.

Second, additional adjustment for eGFR_CKD-EPI_ attenuated partial correlations of serum AGEs and serum dicarbonyls with serum biomarkers of ED and LGI (S1 Fig). The correlation of GO with sThrombomodulin remained above 0.30 (*e*.*g*., Spearman’s ρ 0.34), albeit not statistically significantly. In contrast, the correlation of free CEL with IL-8 (Spearman’s ρ 0.36) became statistically significant. In addition, additional adjustment for fluid overload did not materially change results (S2 Fig).

Third, additional adjustment for eGFR_CKD-EPI_ attenuated the associations of protein-bound CML (beta 0.16 (-0.26; 0.59), *P* = 0.439), free CEL (beta 0.17 (-0.23; 0.58), *P* = 0.390) and protein-bound MG-H1 (beta 0.26 (-0.11; 0.63), *P* = 0.166) with the composite score of LGI. However, 95% confidence intervals were wide. In addition, additional adjustment for fluid overload did not materially change results (results not shown).

Fourth, a correlation matrix of levels of AGEs and dicarbonyls, adjusted for age, sex and diabetes mellitus (S3 Fig) showed positive correlations among free AGEs, among protein-bound AGEs and among dicarbonyls. In addition, the free and protein-bound fractions of AGEs were correlated and there was an extensive correlation among serum AGEs and dicarbonyls. Further, SAF was not correlated with serum AGEs and dicarbonyls.

Fifth, S5 Table shows characteristics of the study population stratified according to the presence of a history of CVD. Logistic regression analyses suggested that higher levels of most of the measured serum AGEs and dicarbonyls were associated with a history of CVD, albeit not statistically significantly (S6 Table). Similarly, in particular sVCAM-1, sThrombomodulin, IL-6 and TNF-α were numerically associated with a history of CVD (S7 Table). Nevertheless, 95% confidence intervals of the odds ratios were (very) wide.

## Discussion

This study in CKD5-ND showed that *higher* levels of several serum AGEs and serum dicarbonyls, but not SAF, correlated with *higher* levels of several individual serum biomarkers of ED and LGI after adjustment for age, sex and diabetes mellitus. However, most correlations and associations were attenuated after additional adjustment for eGFR_CKD-EPI_, which suggests that eGFR_CKD-EPI_ confounds and/or mediates the relation of serum AGEs and dicarbonyls with ED and LGI.

The relations of several serum AGEs and dicarbonyls with sVCAM-1 and sThrombomodulin agree with and extend results of previous studies in CKD [44], CKD5-ND [30] and CKD5-D [24,26], which observed associations of plasma pentosidine [24,30], serum CML [44] and SAF [26] with biomarkers of ED, for example, sVCAM-1 [30,44], endothelin-1 [24], post-occlusive peak reactive hyperemia [44], thermal hyperemia [44] and flow-mediated dilation [26].

In contrast, results of studies in CKD [45], CKD5-ND [20,28,30] and CKD5-D [20–22,25,28,29] which examined the relation of serum AGEs [20–22,30,45] and SAF [25,28,29] with LGI are inconsistent. This may be related to differences in measured AGEs, not distinguishing between free and protein-bound AGEs fractions, which may be divergently affected by kidney function and dialysis treatment [15,31,46]; the use of less accurate analytical techniques to measure AGEs (*i*.*e*., non-specific fluorescent AGEs [20,22], enzyme linked immunosorbent assay (ELISA) or dot blotting antibody methods for measurement of pentosidine [21] and CML [20–22]), differences in selection of biomarkers of LGI (*i*.*e*., CRP [20,22,25,28–30], IL-6 [20,30], TNF-α [20,45], interleukin-1 receptor antagonist [45], and IL-18 [21]), concurrent dialysis treatment and adjustment for potential confounders.

The present study used state-of-the-art tandem mass spectrometry to measure several specific AGEs in a CKD5 population not on dialysis and showed correlations of several serum AGEs and MGO with hs-CRP, SAA and/or IL-6. This combination of serum biomarkers of LGI is consistent as IL-6 stimulates the synthesis of both CRP and SAA [47,48].

In-vitro studies provide additional support for an association of AGEs and dicarbonyls with ED and LGI, as reviewed elsewhere [6,8,10–12]. Potential mechanisms include activation of the NF-κB pathway through RAGE signaling, which increases transcription of endothelial adhesion molecules and pro-inflammatory cytokines; activation of monocytes, increased oxidative stress, reduced bioavailability of nitric oxide, and endothelial cell detachment. Conversely, LGI may stimulate formation of dicarbonyls and AGEs. Indeed, exposing monocytes to TNF-α reduced glyoxalase-1 expression and increased formation of MGO, CML and MG-H1 [13]. Thus, the relation of AGEs and dicarbonyls with LGI and ED, which is closely linked to LGI [49], may be bidirectional.

Importantly, attenuation of the above mentioned correlations by additional adjustment for eGFR_CKE-EPI_ suggested that GFR confounds and/or mediates associations of serum AGEs and serum dicarbonyls with serum biomarkers of ED and LGI. Similar results were observed in a study in CKD3-5 [45]. Indeed, GFR may be a confounder as kidney function decline could lead to accumulation of AGEs and dicarbonyls as well as ED and LGI via independent mechanisms. This is not unlikely given the plethora of metabolic alterations observed in ESRD. However, AGEs and dicarbonyls have been hypothesized to be involved in the development and progression of kidney disease as well [10,50]. Therefore, AGEs and dicarbonyls may stimulate ED and LGI *via* kidney function decline. Thus, correlations and associations adjusted for eGFR may be underestimated (*i*.*e*., overadjustment bias [51]).

Whether confounding or mediation, preservation of kidney function may be worthwhile to prevent accumulation of AGEs, dicarbonyls and biomarkers of ED and LGI in ESRD.

The results of this study suggest that SAF and serum AGEs measure different processes in ESRD. Indeed, the relation of serum AGEs with serum biomarkers of ED and LGI was not accompanied by similar relations of SAF. In addition, SAF was not correlated with serum AGEs in this CKD5-ND cohort and results of the limited studies on the correlation between SAF and serum AGEs are inconsistent, even for the fluorophore pentosidine [52–54].

The use of composite scores of ED and LGI may increase statistical efficiency and reduce the influence of biological and analytical variability [40]. However, associations with composite scores are attenuated if associations of individual serum biomarkers (unexpectedly) differ with regard to their direction and strength.

Indeed, the specificity of the biomarkers on the panel which was used in this study to assess ED and LGI varies, as these biomarkers may be also expressed on non-endothelial and non-inflammatory cells, or may only be expressed under specific circumstances. For example, P-selectin may be expressed on activated thrombocytes as well as endothelial cells[55,56], and ICAM-3 is primarily expressed on leukocytes instead of endothelial cells[57], unless these are located in benign or malignant tumors [58]. This could explain the absence of a positive correlation of AGEs and dicarbonyls with sP-selectin and sICAM-3 as well as the more convincing results of the correlation analyses than the analyses with composite scores for the other individual serum biomarkers of ED. Therefore, we consider the analyses with composite scores as complementary to those with individual biomarkers.

The key strength of this study was the combination of multiple serum AGEs, serum dicarbonyls, SAF and serum biomarkers of ED and LGI to cover the heterogeneous family of AGEs and the concepts of ED and LGI. In addition, restricting the study population to individuals with CKD5-ND allowed studying the uremic milieu *per se* as it precludes possible confounding by different renal replacement modalities.

This study also had some limitations. First, the cross-sectional design limited causal inferences. Second, the relatively small sample size of the study restricted study power. This was reflected by the wide confidence intervals in the regression analyses. In addition, the sample size did not allow for an adequate analysis of the association of the measured biomarkers with clinical manifestations of CVD. Third, three outliers likely affected the regression analyses. However, the medical history of two of these outliers provided a plausible explanation for their deviating behavior (*i*.*e*., an underlying infection or inflammatory response). In addition, exclusion of outliers mostly affected the precision and not the direction or strength of the associations reported. Fourth, chance findings due to multiple testing cannot be excluded. Nevertheless, the analyses showed a consistent pattern of correlations of most serum AGEs and serum dicarbonyls with sVCAM-1 and sThrombomodulin as serum biomarkers of ED and with hs-CRP, SAA and IL-6 as serum biomarkers of LGI, albeit not all statistically significantly. In addition, the number of statistically significant correlations (*i*.*e*., 14) exceeded the number of statistically significant correlations expected by chance alone (*i*.*e*., 0.05 * 110 = 5.5). Fifth, for practical reasons, not all participants were in the fasting state. However, this may confound levels of serum dicarbonyls, which rise following a meal [59]. Sixth, data on pentosidine were lacking. Such data may have provided additional details on the discrepancy between serum AGEs and SAF as pentosidine is a fluorescent AGE [37].

In conclusion, in individuals with CKD5-ND, *higher* levels of serum AGEs and serum dicarbonyls were related to ED and LGI after adjustment for age, sex and diabetes mellitus, but not after additional adjustment for eGFR. An association of AGEs and dicarbonyls with ED and LGI may provide a mechanism for the increased CVD risk in ESRD. The role of eGFR as a confounder and/or mediator in the associations reported warrants further study and experimental studies may further unravel this issue.

## Supporting information

S1 Methods (DOCX)

S2 Methods (DOC)

S1 Table Association of estimated glomerular filtration rate (eGFR_CKD-EPI_) with advanced glycation endproducts and dicarbonyls. (DOCX)

S2 Table Association of estimated glomerular filtration rate (eGFR_CKD-EPI_) with serum biomarkers of endothelial dysfunction and low-grade inflammation. (DOCX)

S3 Table Characteristics of outliers. (DOCX)

S4 Table Associations of advanced glycation endproducts and dicarbonyls with endothelial and low-grade inflammation stratified by dialysis status after exclusion of outliers. (DOCX)

S5 Table Population characteristics stratified by history of cardiovascular disease. (DOCX)

S6 Table Associations of advanced glycation endproducts and dicarbonyls with history of cardiovascular disease. (DOCX)

S7 Table Associations of serum biomarkers of endothelial dysfunction and low-grade inflammation with history of cardiovascular disease. (DOCX)

S1 Fig Partial Spearman’s rank correlations of advanced glycation endproducts and dicarbonyls with individual serum biomarkers of endothelial dysfunction and low-grade inflammation after additional adjustment for kidney function. (DOCX)

S2 Fig Partial Spearman’s rank correlations of advanced glycation endproducts and dicarbonyls with individual serum biomarkers of endothelial dysfunction and low-grade inflammation after additional adjustment for fluid overload. (DOCX)

S3 Fig Partial Spearman’s rank correlations among serum advanced glycation endproducts, serum dicarbonyls and skin autofluorescence. (DOCX)
