# Supplementary material for: Relations of advanced glycation endproducts and dicarbonyls with endothelial dysfunction and low-grade inflammation in individuals with end-stage renal disease in the transition to renal replacement therapy: A cross-sectional observational study
Source: PLoS One. 2019 Aug 13;14(8):e0221058. doi: 10.1371/journal.pone.0221058 (PMC6692010; doi:10.1371/journal.pone.0221058)
Supplement: S1 Methods — (DOCX) [file pone.0221058.s001.docx]

**S1 Methods**

***In- and exclusion criteria of the individual studies***

Incident dialysis patients were individuals with ESRD who would start with dialysis within one month. Measurements were performed maximum four weeks prior to the first dialysis session. Exclusion criteria for incident dialysis patients were: acute start of dialysis treatment, active symptomatic coronary artery disease or cardiac failure New York Heart Association (NYHA) class III or IV, active malignancies, active infections, and inability to provide informed consent. Incident dialysis patients were recruited from the following dialysis centers in the South East of the Netherlands: Maastricht University Medical Center+ Maastricht, Catharina Hospital Eindhoven, Viecuri Hospital Venlo, Zuyderland Medical Centre Sittard, and St. Laurentius Hospital Roermond (Ethical Committee study number NL33129.068.10). This study was conducted between February 2012 and July 2017.

Kidney transplant recipients were individuals with ESRD receiving a living donor kidney transplant, age ≥18 years, and ability to provide informed consent. These individuals were recruited from the pre-transplantation clinic at the Maastricht University Medical Center+ in the Netherlands (Ethical Committee study number NL43381.068.13). Baseline measurements were performed within five days before kidney transplantation, with the exception of one patient who was examined five weeks before kidney transplantation. This study was conducted between October 2013 and January 2018.
