# Supplementary material for: Relations of advanced glycation endproducts and dicarbonyls with endothelial dysfunction and low-grade inflammation in individuals with end-stage renal disease in the transition to renal replacement therapy: A cross-sectional observational study
Source: PLoS One. 2019 Aug 13;14(8):e0221058. doi: 10.1371/journal.pone.0221058 (PMC6692010; doi:10.1371/journal.pone.0221058)
Supplement: S2 Methods — (DOC) [file pone.0221058.s002.doc]

**S2 Methods**

**Determination of protein-bound and free AGEs**

This document provides a description and validation of the applied method to determine plasma or serum levels of free and protein-bound *N*^∈^-(carboxymethyl)lysine (CML), *N*^∈^-(carboxyethyl)lysine (CEL) and *N*_δ_-(5-hydro-5-methyl-4-imidazolon-2-yl)ornithine (MG-H1). The primary validation study as described below was performed with plasma. Its results also apply to serum (results not shown).

***Materials***

Boric acid, sodium borohydride, trifluoroacetic acid (TFA) and chloroform were obtained from Sigma-Aldrich (Zwijndrecht, the Netherlands). Butanol and methanol were obtained from Baker (Deventer, the Netherlands). Sodium hydroxide and hydrochloric acid were obtained from Merck (Darmstadt, Germany). Water and acetonitrile were obtained from Biosolve BV (Valkenswaard, the Netherlands). CML (99%), [^2^H_2_]-CML (95.7%), CEL (98.6%), [^2^H_4_]-CEL, MG-H1 (92.6%) and [^2^H_3_]-MG-H1 (98.7%) were obtained from PolyPeptide (Strassbourg, France). All other reagents and solvents were of analytical or ULC/MS grade.

***Sample preparation protein-bound AGEs***

Whole blood was collected in sterile EDTA tubes (for the validation study). After centrifugation plasma was aspirated and stored at -80°C until analysis. Before analysis, plasma samples where thawed and mixed thoroughly. Twenty-five µL of plasma was mixed with 50 µL water in a 4 mL reaction vial. After addition of 200 µL of 100 mmol/L sodium borohydride dissolved in 200 mmol/L borate buffer (pH 9.2), the samples were incubated at room temperature for 2 hours. Samples were mixed and subsequently deproteinized with 1000 µL cold (4°C) TFA. After centrifugation (4300 g, 4°C, 20 min) the supernatant was carefully removed with a Pasteur pipette.

For validation experiments 25 µL of a standard solution (six point calibration curve; 5250-0 nmol/L CML, 6250-0 nmol/L CEL and 14749-0 nmol/L MG-H1) was added. Samples were then hydrolyzed by adding 500 µL 6 N HCl and incubated for 20 hours at 90°C. After hydrolysis 40 µL hydrolysate and 20 µL internal standard (containing 1432 nmol/L [^2^H_2_]-CML, 1378 nmol/L [^2^H_4_]-CEL and 1322 nmol/L [^2^H_3_]-MG-H1) was mixed in a reaction vial. This mixture was evaporated to dryness under a stream of nitrogen gas at 70°C and subsequently derivatized in 100 µL 1-butanol:HCl (3:1, v/v) for 90 minutes at 70°C. Samples were then evaporated to dryness under nitrogen and redissolved in 200 µL water.

***Sample preparation free AGEs***

Twenty-five μL of internal standard (containing 716 nmol/L [^2^H_2_]-CML, 689 nmol/L [^2^H_4_]-CEL and 661 nmol/L [^2^H_3_]-MG-H1) was mixed with 50 μL plasma. Samples were mixed thoroughly and subsequently deproteinized with 600 μL of a mixture of methanol and acetonitrile (1:3, by volume) and centrifuged at 14000 rpm for 20 min at room temperature. The supernatant was transferred to a reaction vial and further treated as described for the protein-bound AGEs.

***UPLC tandem MS analysis AGEs***

Derivatized CML, CEL and MG-H1 were analyzed by ultraperformance liquid chromatography (Acquity UPLC, Waters, Milford, USA) and detected in ESI positive multiple reaction monitoring (MRM) mode using a Xevo TQ MS (Waters, Milford, USA). Derivatives were separated on a reversed-phase C18 column (Acquity UPLC BEH C18, 50 x 2.1 mm, 1.7 µm) with a linear gradient of 5 mmol/L ammonia and acetonitril at 48°C. The flow rate was 800 µL/min and the injection volume was 2 µL. Quantification of CML, CEL and MG-H1 was performed by calculating the peak area ratio of each unlabeled peak area to the corresponding internal standard peak area. The MRM transitions for CML, CEL and MG-H1 were respectively 317.1>186.1, 331.1>186.1 and 285.1>172.1. The MRM transitions for the internal standards [^2^H_2_]-CML, [^2^H_4_]-CEL, and [^2^H_3_]-MG-H1 were respectively 319.1>186.1, 335.1>190.1 and 288.1>172.1. Electrospray ionization was done at a capillary voltage of 0.5 kV, a source temperature of 150°C and a desolvation temperature of 600°C. For qualitative and quantitative analysis, Masslynx software (V4.1, SCN 644, Waters) was used.

***Method validation AGEs***

Linearity was determined by adding standard solution of CML, CEL and MG-H1 to water and five different plasma samples. For protein-bound AGEs in plasma, a six-point calibration curve was prepared: CML (0-5250 nmol/L), CEL (0-6250 nmol/L) and MG-H1 (0-14750 nmol/L). For free AGEs in plasma, a six-point calibration curve was prepared: CML (0-525 nmol/L), CEL (0-625 nmol/L) and MG-H1 (0-1475 nmol/L). The peak area ratio of CML, CEL and MG-H1 multiplied by the concentration of each corresponding internal standard were plotted as a function of the concentration. For evaluation of inter- and intra-assay variation, a pooled EDTA plasma sample was analyzed on 10 different days (inter-assay) and 10 times on the same day (intra-assay).

***Acid stability of AGEs***

For protein-bound AGE analysis an acid hydrolysis is needed. To investigate acid stability of CML, CEL and MG-H1 a six-point calibration curve was prepared as described in the method validation section and subsequently hydrolyzed. This calibration curve was compared to a calibration curve without acid hydrolysis. Moreover, reproducibility of protein-bound AGE analysis was tested by the analysis of 12 different plasma samples on two different days.

***Results***

**UPLC tandem MS analysis AGEs**

A representative chromatogram of free AGEs in an EDTA pool plasma, protein-bound AGEs in an EDTA pool plasma and a standard in water is shown in figure 1 A, B and C. Retention times of CML, CEL and MG-H1 were 9.05, 9.70 and 3.71 minutes, respectively, and the corresponding stable isotopes were eluted at 9.03, 9.68 and 3.69, respectively.

| 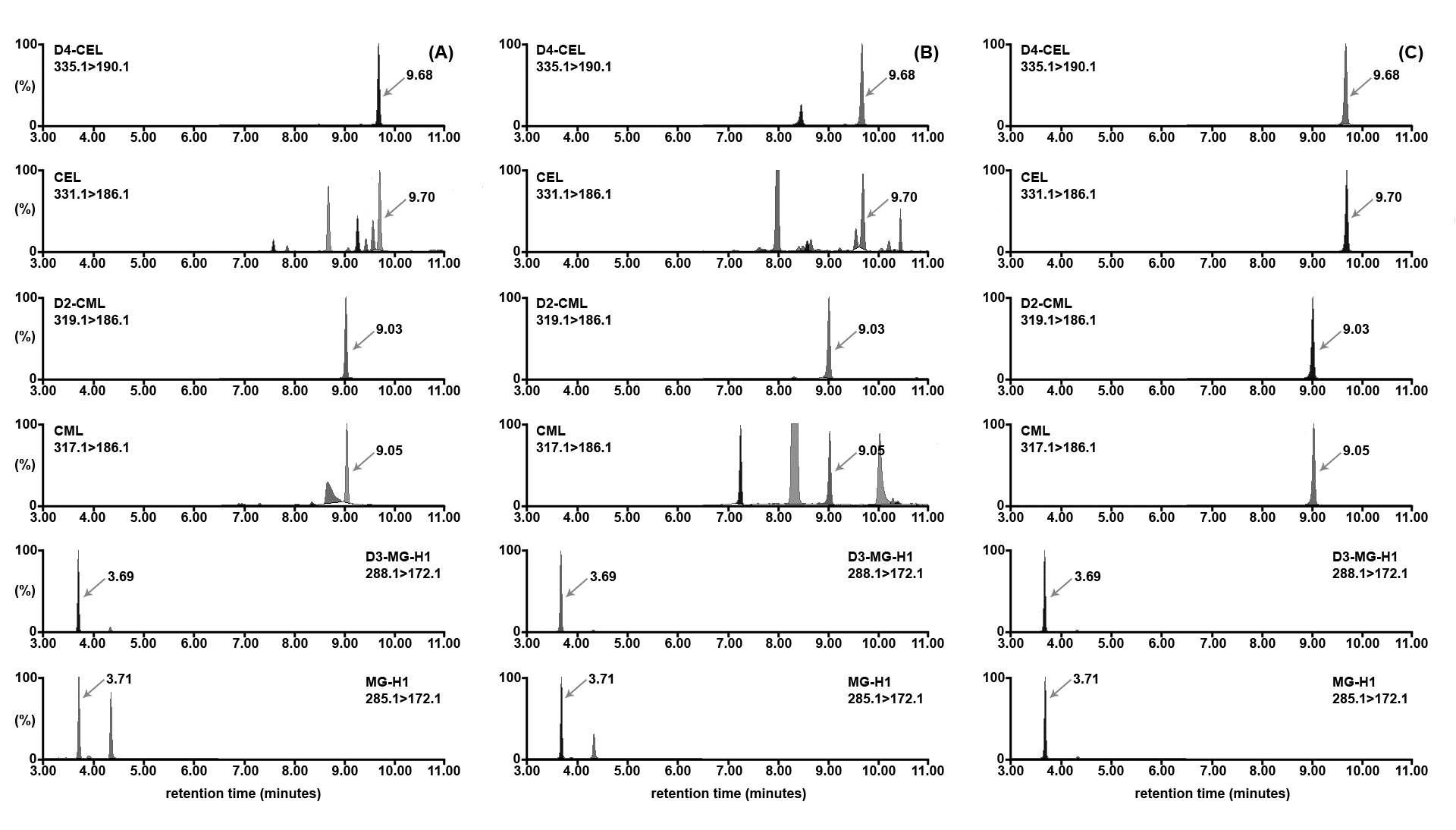 |
| --- |
| **Figure 1.** Representative chromatograms of a standard solution of AGEs (C), an EDTA plasma sample of free AGEs (A) and an EDTA plasma sample of protein-bound AGEs (B). The elution of the AGEs are indicated with an arrow mark and corresponding retention time. Concentrations of CML, CEL and MG-H1 in the standard solution (C) were 5250, 6250 and 14750 nmol/L; in the EDTA plasma free form (A) were 65.7, 44.6 and 329.0 nmol/L; and in the EDTA plasma protein-bound form (B) were 3269, 926 and 4354 nmol/L, respectively. |

**Method validation AGEs**

The calibration curves for CML, CEL and MG-H1 were linear over the described concentration ranges (r^2^>0.99) in both water and plasma matrix. Mean slope (response factor) for protein-bound CML, CEL and MG-H1 as tested in 5 different matrices were respectively 1.106 (CV, 4.8%), 1.401 (CV, 6.2%) and 0.780 (CV, 3.3%). Mean slope for free CML, CEL and MG-H1 as tested in 8 different matrices were respectively 1.050 (CV, 10.0%), 1.463 (CV, 3.1%) and 1.010 (CV, 3.8%).

Inter- and intra-assay variation as determined by replicate analysis of a plasma sample is given in table 1.

|  | **Intra-assay variation** | | | | **Inter-assay variation** | | | |
| --- | --- | --- | --- | --- | --- | --- | --- | --- |
|  | **Protein-bound (n=10)** | | **Free (n=8)** | | **Protein-bound (n=23)** | | **Free (n=8)** | |
| **Compound** | **Mean (SD) nmol/L** | **CV %** | **Mean (SD) nmol/L** | **CV %** | **Mean (SD) nmol/L** | **CV %** | **Mean (SD) nmol/L** | **CV %** |
| CML  CEL  MG-H1 | 3023 (61)  1447 (53)  1307 (69) | 2.0  3.7  5.3 | 66.1 (1.8)  46.9 (1.7)  348.4 (13.0) | 2.8  3.7  3.7 | 3072 (161)  1813 (195)  1230 (107) | 5.3  10.7  8.7 | 70.6 (5.0)  43.5 (2.8)  337.5 (17.1) | 7.1  6.4  5.1 |

**Table 1.** Intra- and inter-assay variation of protein-bound and free AGEs as determined by replicate analysis of a plasma sample.

The lower limits of quantification (s/N=10) on column for protein-bound CML, CEL and MG-H1 were 4, 4, and 7 fmol, corresponding to a concentration of 200, 200 and 340 nmol/L. The lower limits of quantification (s/N=10) on column for free CML, CEL and MG-H1 were 4, 3 and 6 fmol, corresponding to a concentration of 8.5, 4.9 and 11.8 nmol/L.

**Acid stability of AGEs**

The calibration curves for CML, CEL and MG-H1, as tested in water after acid hydrolysis, were compared to the calibration curves without acid hydrolysis. The slope for CML, CEL and MG-H1 as tested in water, without acid hydrolysis, was 1.18, 1.59 and 1.03, respectively. For CML and CEL no differences were observed between the mean slopes of the calibration curve with or without acid hydrolysis (figure 2).

For MG-H1, however, a decrease of the mean slope from 1.03 to 0.70 was observed after acid hydrolysis (figure 2B.3). Thus, under these standard conditions we found a recovery of MG-H1 of ~70%.

| 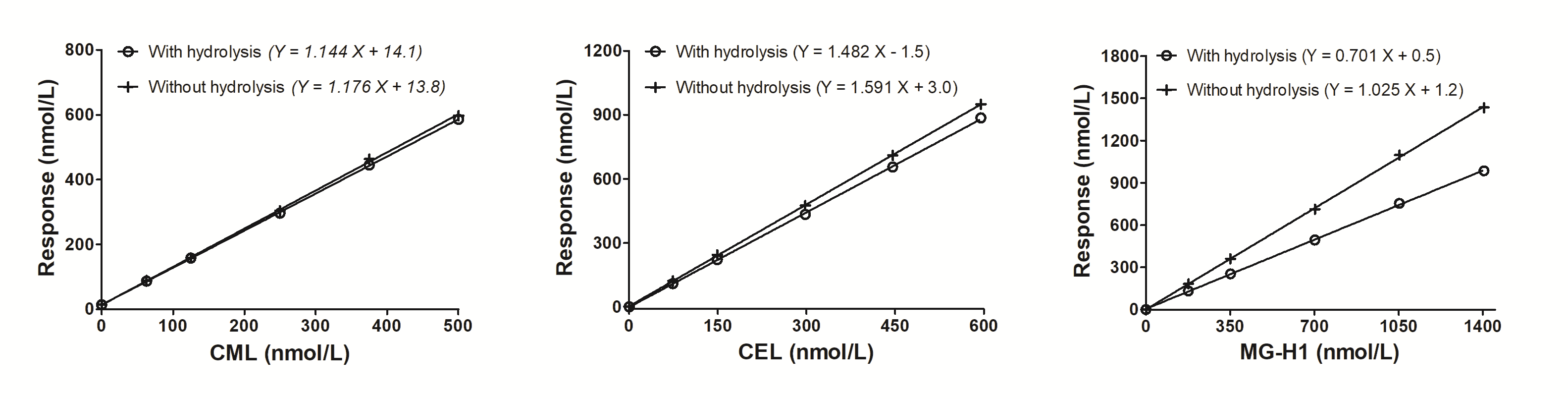 |
| --- |
| **Figure 2.** Calibration curves in water with and without hydrolysis. |

**Reproducibility of protein-bound AGEs in plasma**

Twelve different plasma samples were analyzed on two different days (analysis on day 1 and repeated analysis after 4 days). Perfect correlation (r^2^>0.90) and reproducibility (>80%) was found for protein-bound CML, CEL and MG-H1 in plasma (figure 3).


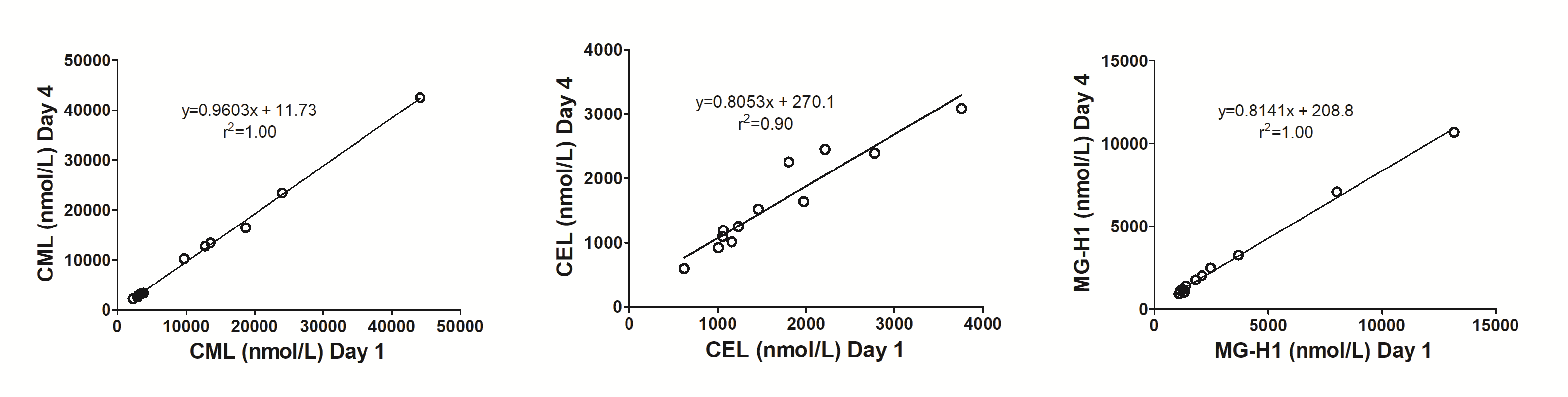


| **Figure 3.** Correlation of repeated analysis of protein-bound AGEs in plasma. |
| --- |

**Additional notes related to present study**

For the present study, serum was collected in 5 mL BD Vacutainer® SST^TM^ II advance tubes. Intra-assay coefficients of variation as determined by replicate analysis of a serum sample (n=8) and inter-assay coefficients of variation as determined by replicate analysis of a plasma sample on different days (n=9) for free AGEs were 3.2% and 8.1% for free CML, 3.6% and 8.0% for free CEL, 3.3% and 4.2% for free MG-H1. Intra-assay coefficients of variation as determined by replicate analysis of a serum sample (n=10) and inter-assay coefficients of variation as determined by replicate analysis of a plasma sample on different days (n=23) for protein-bound AGEs were 2.0% and 5.3% for protein-bound CML, 3.7% and 10.7% for protein-bound CEL, 5.3% and 8.7% for protein-bound MG-H1.
