# Supplementary material for: Relations of advanced glycation endproducts and dicarbonyls with endothelial dysfunction and low-grade inflammation in individuals with end-stage renal disease in the transition to renal replacement therapy: A cross-sectional observational study
Source: PLoS One. 2019 Aug 13;14(8):e0221058. doi: 10.1371/journal.pone.0221058 (PMC6692010; doi:10.1371/journal.pone.0221058)
Supplement: S1 Table — (DOCX) [file pone.0221058.s003.docx]

S1 Table. Association of estimated glomerular filtration rate (eGFR_CKD-EPI_) with advanced glycation endproducts and dicarbonyls

| Biomarker | Ratio (95%CI) | *P* value |
| --- | --- | --- |
| CML_free_ | 1.08 (1.02; 1.14) | 0.008 |
| CML_protein-bound_ | 1.07 (1.04; 1.10) | < 0.001 |
| CEL_free_ | 1.07 (1.01; 1.13) | 0.028 |
| CEL_protein-bound_ | 1.03 (0.99; 1.07) | 0.097 |
| MG-H1_free_ | 1.08 (1.01; 1.15) | 0.021 |
| MG-H1_protein-bound_ | 1.05 (1.02; 1.08) | < 0.001 |
| GO | 1.04 (1.01; 1.08) | 0.013 |
| MGO | 1.04 (1.00; 1.09) | 0.030 |
| 3-DG | 1.02 (0.99; 1.05) | 0.221 |
| SAF | 1.00 (0.98; 1.02) | 0.709 |

Ratios represent the ratio of (geometric mean) levels of the biomarkers per 1 mL/min/1.73m^2^ lower eGFR.

All analyses are adjusted for age, sex and diabetes mellitus.

Abbreviations: 3-DG, 3-deoxyglucosone; CEL, *N*^∈^-(carboxyethyl)lysine; CI, confidence interval; CML, *N*^∈^-(carboxymethyl)lysine; GO, glyoxal; MG-H1, *N*_δ_-(5-hydro-5-methyl-4-imidazolon-2-yl)ornithine; MGO, methylglyoxal; SAF, skin autofluorescence.

Analyses based on n = 42 for serum AGEs, n = 38 for dicarbonyls, and n = 40 for skin autofluorescence.
