# Supplementary material for: Relations of advanced glycation endproducts and dicarbonyls with endothelial dysfunction and low-grade inflammation in individuals with end-stage renal disease in the transition to renal replacement therapy: A cross-sectional observational study
Source: PLoS One. 2019 Aug 13;14(8):e0221058. doi: 10.1371/journal.pone.0221058 (PMC6692010; doi:10.1371/journal.pone.0221058)
Supplement: S2 Table — (DOCX) [file pone.0221058.s004.docx]

S2 Table. Association of estimated glomerular filtration rate (eGFR_CKD-EPI_) with serum biomarkers of endothelial dysfunction and low-grade inflammation

| Serum biomarkers | Ratio (95%CI) | *P* value |
| --- | --- | --- |
| sVCAM-1 | 1.02 (0.99; 1.05) | 0.160 |
| E-selectin | 1.01 (0.96; 1.06) | 0.666 |
| P-selectin | 0.99 (0.95; 1.03) | 0.510 |
| SThrombomodulin | 1.02 (0.99; 1.05) | 0.114 |
| sICAM-1 | 1.01 (0.98; 1.03) | 0.650 |
| sICAM-3 | 1.00 (0.96; 1.04) | 0.977 |
| hs-CRP | 1.17 (1.02; 1.34) | 0.029 |
| SAA | 1.25 (1.08; 1.45) | 0.005 |
| IL-6 | 1.06 (0.98; 1.15) | 0.132 |
| IL-8 | 0.99 (0.92; 1.06) | 0.691 |
| TNF-α | 0.99 (0.95; 1.02) | 0.492 |

Ratios represent the ratio of (geometric mean) levels of the serum biomarkers per 1 mL/min/1.73m^2^ lower eGFR.

Betas represent the differences in Z-scores for endothelial dysfunction and low-grade inflammation (expressed as standard deviations) per 1 mL/min/1.73m^2^ lower eGFR.

All analyses are adjusted for age, sex and diabetes mellitus.

Abbreviations: hs-CRP, high-sensitivity C-reactive protein; IL-6, interleukin 6; IL-8, interleukin 8; SAA, serum amyloid A; sE-selectin, soluble E-selectin; sICAM-1, soluble intercellular adhesion molecule 1; sICAM-3, soluble intercellular adhesion molecule 3; sP-selectin, soluble P-selectin; sThrombomodulin, soluble Thrombomodulin; sVCAM-1, soluble vascular cell adhesion molecule 1; TNF-α, tumor necrosis factor alpha.

Analyses based on n = 42.
