# Supplementary material for: Relations of advanced glycation endproducts and dicarbonyls with endothelial dysfunction and low-grade inflammation in individuals with end-stage renal disease in the transition to renal replacement therapy: A cross-sectional observational study
Source: PLoS One. 2019 Aug 13;14(8):e0221058. doi: 10.1371/journal.pone.0221058 (PMC6692010; doi:10.1371/journal.pone.0221058)
Supplement: S3 Table — (DOCX) [file pone.0221058.s005.docx]

S3 Table. Characteristics of outliers

|  | Regression analyses for which the participant is an outlier | | |
| --- | --- | --- | --- |
|  | ED (residuals < - 2SD) | ED and LGI (residuals > +2SD) | LGI (residuals > +2SD) |
|  | High AGEs and dicarbonyls/  Low biomarkers of ED | Low AGEs and dicarbonyls/  High biomarkers of ED and LGI | Low AGEs/  High biomarkers of LGI |
| *Clinical characteristics* | |  |  |
| Age (years) | 55 | 69 | 42 |
| Sex | Man | Man | Man |
| Origin of end-stage renal disease | Polycystic kidney disease | Polycystic kidney disease | Unknown |
| History of KTx | No | Yes | No |
| First future treatment modality | Peritoneal dialysis | Hemodialysis | Kidney transplantation |
| eGFR_CKD-EPI_ (mL/min/1.73m^2^) | 4.6 | 6.2 | 10.3 |
| Residual urine output | Yes | Yes | Yes |
| Residual urine output (mL/24h) | Not available | 2250 | 2000 |
| Diabetes mellitus | No | No | No |
| Cardiovascular disease | No | No | No |
| BMI (kg/m^2^) | 23.3 | 26.8 | 24.8 |
| Fluid overload (L) | 0.7 | 4.7 | 0.9 |
| SBP (mmHg) | 112 | 180 | 116 |
| DBP (mmHg) | 87 | 96 | 79 |
| *Advanced glycation endproducts and dicarbonyls* | |  |  |
| CML_free_ (nmol/L) | 1954.8 | 1304.2 | 571.2 |
| CML­_protein-bound_ (nmol/mmol lysine) | 418.3 | 240.3 | 166.0 |
| CEL_free_ (nmol/L) | 1159.6 | 812.6 | 578.6 |
| CEL_protein-bound_ (nmol/mmol lysine) | 76.9 | 65.3 | 38.1 |
| MG-H1_free_ (nmol/L) | 4116.1 | 2062.0 | 1712.6 |
| MG-H1­_protein-bound_ (nmol/mmol lysine) | 96.7 | 66.9 | 54.5 |
| GO (nmol/L) | 2222.4 | 1334.0 | 1034.7 |
| MGO (nmol/L) | 1477.4 | 1089.6 | 1452.0 |
| 3-DG (nmol/L) | 2297.3 | 1409.1 | 801.0 |
| SAF (AU) | 3.9 | 4.3 | 2.5 |
| *Serum biomarkers of endothelial dysfunction and low-grade inflammation* | | | |
| sVCAM-1 (μg/L) | 676.0 | 1788.0 | 696.0 |
| E-selectin (μg/L) | 7.0 | 19.0 | 9.9 |
| P-selectin (μg/L) | 25.9 | 43.5 | 130.1 |
| SThrombomodulin (μg/L) | 8.2 | 13.8 | 9.3 |
| sICAM-1 (μg/L) | 319.0 | 773.0 | 412.0 |
| sICAM-3 (μg/L) | 0.6 | 1.6 | 1.0 |
| hs-CRP (mg/L) | 1.2 | 123.9 | 6.2 |
| SAA (mg/L) | 5.9 | 263.2 | 15.7 |
| IL-6 (ng/L) | 1.7 | 5.3 | 12.3 |
| IL-8 (ng/L) | 18.4 | 21.7 | 338.0 |
| TNF-α (ng/L) | 3.7 | 6.1 | 19.5 |
| *Explanation for outlier status* | | | |
| Medical record | No relevant data | Recurring infected liver cysts | Hospital admission for fever due to viral infection two weeks before study participation |

Abbreviations: 3-DG, 3-deoxyglucosone; AU, arbitrary units; BMI, body mass index; CEL, *N*^∈^(carboxyethyl)lysine; CML, *N*^∈^(carboxymethyl)lysine; DBP, diastolic blood pressure; eGFR_CKD-EPI_, estimated glomerular filtration rate based on the creatinine CKD-EPI equation; GO, glyoxal; hs-CRP, high-sensitivity C-reactive protein; IL-6, interleukin 6; IL-6, interleukin 8; KTx, kidney transplantation; MG-H1, *N*_δ_(5-hydro-5-methyl-4-imidazolon-2-yl)ornithine; MGO, methylglyoxal; SAA, serum amyloid A; SAF, skin autofluorescence; SBP, systolic blood pressure; sE-selectin, soluble E-selectin; sICAM-1, soluble intercellular adhesion molecule 1; sP-selectin, soluble P-selectin; sThrombomodulin, soluble Thrombomodulin; sVCAM-1, soluble vascular cell adhesion molecule 1; TNF-α, tumor necrosis factor alpha.
