# Supplementary material for: Relations of advanced glycation endproducts and dicarbonyls with endothelial dysfunction and low-grade inflammation in individuals with end-stage renal disease in the transition to renal replacement therapy: A cross-sectional observational study
Source: PLoS One. 2019 Aug 13;14(8):e0221058. doi: 10.1371/journal.pone.0221058 (PMC6692010; doi:10.1371/journal.pone.0221058)
Supplement: S4 Table — (DOCX) [file pone.0221058.s006.docx]

S4 Table. Associations of advanced glycation endproducts and dicarbonyls with endothelial and low-grade inflammation stratified by dialysis status after exclusion of outliers

|  |  | Endothelial dysfunction* | | Low-grade inflammation* | |
| --- | --- | --- | --- | --- | --- |
| Biomarker | Model | Beta (95%CI) | *P* value | Beta (95%CI) | *P* value |
| CML_free_ | 1 | 0.08 (-0.21; 0.37) | 0.599 | 0.13 (-0.14; 0.41) | 0.253 |
|  | 2 | 0.08 (-0.23; 0.38) | 0.621 | 0.17 (-0.12; 0.46) | 0.235 |
| CML­­_protein-bound_ | 1 | 0.23 (-0.06; 0.52) | 0.110 | 0.29 (0.03; 0.54) | 0.029 |
|  | 2 | 0.22 (-0.09; 0.54) | 0.159 | 0.26 (-0.01; 0.53) | 0.063 |
| CEL­­_free_ | 1 | 0.17 (-0.11; 0.45) | 0.223 | 0.26 (0.00; 0.51) | 0.052 |
|  | 2 | 0.19 (-0.12; 0.49) | 0.222 | 0.30 (0.02; 0.57) | 0.034 |
| CEL_protein-bound_ | 1 | 0.06 (-0.23; 0.34) | 0.686 | 0.11 (-0.16; 0.38) | 0.421 |
|  | 2 | 0.08 (-0.21; 0.38) | 0.570 | 0.16 (-0.12; 0.44) | 0.255 |
| MG-H1_free_ | 1 | 0.04 (-0.25; 0.32) | 0.797 | 0.17 (-0.10; 0.44) | 0.204 |
|  | 2 | 0.05 (-0.25; 0.35) | 0.744 | 0.19 (-0.09; 0.46) | 0.175 |
| MG-H1_protein-bound_ | 1 | 0.15 (-0.13; 0.44) | 0.286 | 0.29 (0.04; 0.55) | 0.026 |
|  | 2 | 0.15 (-0.15; 0.45) | 0.319 | 0.28 (0.02; 0.53) | 0.033 |
| GO | 1 | 0.35 (0.06; 0.63) | 0.019 | 0.35 (0.06; 0.64) | 0.018 |
|  | 2 | 0.36 (0.02; 0.70) | 0.039 | 0.39 (0.06; 0.72) | 0.023 |
| MGO | 1 | 0.24 (-0.05; 0.54) | 0.106 | 0.25 (-0.04; 0.53) | 0.088 |
|  | 2 | 0.21 (-0.12; 0.54) | 0.206 | 0.26 (-0.04; 0.56) | 0.084 |
| 3-DG | 1 | 0.25 (-0.04; 0.55) | 0.090 | 0.23 (-0.12; 0.57) | 0.188 |
|  | 2 | 0.28 (-0.13; 0.68) | 0.177 | 0.09 (-0.38; 0.57) | 0.692 |
| SAF | 1 | 0.23 (-0.05; 0.51) | 0.103 | 0.15 (-0.15; 0.46) | 0.318 |
|  | 2 | 0.23 (-0.11; 0.58) | 0.182 | 0.01 (-0.33; 0.36) | 0.932 |

Betas represent the standardized difference in Z scores of endothelial dysfunction and low-grade inflammation per 1 standard deviation higher transformed levels of serum advanced glycation endproducts and serum dicarbonyls, and per 1 standard deviation higher skin autofluorescence.

All serum advanced glycation endproducts and serum dicarbonyls were natural log transformed, except for free *N*^∈^(carboxymethyl)lysine (square root transformation), protein-bound MG-H1 (inverse transformation) and 3-deoxyglucosone (inverse transformation). In addition, skin autofluorescence was analyzed on its original scale.

Model 1: unadjusted analyses, model 2: adjusted for age, sex and diabetes mellitus.

Abbreviations: 3-DG, 3-deoxyglucosone; CEL, *N*^∈^(carboxyethyl)lysine; CML, *N*^∈^(carboxymethyl)lysine; GO, glyoxal; MG-H1, *N*_δ_(5-hydro-5-methyl-4-imidazolon-2-yl)ornithine; MGO, methylglyoxal; NA, not applicable; SAF, skin autofluorescence.

* Analyses based on n = 40 for serum AGEs, n = 36 for serum dicarbonyls and n = 36 for skin autofluorescence.
