# Supplementary material for: Relations of advanced glycation endproducts and dicarbonyls with endothelial dysfunction and low-grade inflammation in individuals with end-stage renal disease in the transition to renal replacement therapy: A cross-sectional observational study
Source: PLoS One. 2019 Aug 13;14(8):e0221058. doi: 10.1371/journal.pone.0221058 (PMC6692010; doi:10.1371/journal.pone.0221058)
Supplement: S5 Table — (DOCX) [file pone.0221058.s007.docx]

S5 Table. Population characteristics stratified by history of cardiovascular disease

|  |  |  |  |
| --- | --- | --- | --- |
|  | Study population | Without cardiovascular disease | With cardiovascular disease |
|  | (n = 43) | (n = 29) | (n = 14) |
| *Clinical characteristics* | | | |
| Age (years) | 58.8 ±13.9 | 53.8 ±12.5 | 69.2 ±10.8 |
| Men | 29 (67.4%) | 18 (62.1%) | 11 (78.6%) |
| Origin of end-stage renal disease: |  |  |  |
| Nephrosclerosis | 6 (14.0%) | 2 (6.9%) | 4 (28.6%) |
| Glomerulosclerosis | 2 (4.7%) | 1 (3.5%) | 1 (7.1%) |
| Hypertensive nephropathy | 2 (4.7%) | 2 (6.9%) | 0 (0.0%) |
| Renovascular disease | 0 (0.0%) | 0 (0.0%) | 0 (0.0%) |
| Diabetic nephropathy | 2 (4.7%) | 2 (6.9%) | 0 (0.0%) |
| Polycystic kidney disease | 11 (25.6%) | 8 (27.6%) | 3 (21.4%) |
| IgA nephropathy | 2 (4.7%) | 2 (6.9%) | 0 (0.0%) |
| Glomerulonephritis | 4 (9.3%) | 4 (13.8%) | 0 (0.0%) |
| Nephrotic syndrome | 6 (14.0%) | 3 (10.3%) | 3 (21.4%) |
| Other | 1 (2.3%) | 1 (3.5%) | 0 (0.0%) |
| Unknown | 7 (16.3%) | 4 (13.8%) | 3 (21.4%) |
| History of KTx | 8 (18.6%) | 6 (20.7%) | 2 (14.3%) |
| First future treatment modality |  |  |  |
| HD | 19 (44.1%) | 11 (47.8%) | 8 (57.1%) |
| PD | 18 (41.9%) | 12 (52.2%) | 6 (42.9%) |
| Preemptive KTx | 6 (14.0%) | 6 (20.7%) | 0 (0.0%) |
| Non-preemptive KTx | 0 (0.0%) | 0 (0.0%) | 0 (0.0%) |
| eGFR_CKD-EPI_ (mL/min/1.73m^2^)* | 8.6 ±3.1 | 8.8 ±3.1 | 8.4 ±3.3 |
| Residual urine output* | 38 (100%) | 26 (100%) | 12 (100%) |
| Residual urine output (mL/24h)* | 2000 [1700-2296] | 2000 [1763-1476] | 2100 [1476-2348] |
| Diabetes mellitus | 5 (11.6%) | 2 (6.9%) | 3 (21.4%) |
| Cardiovascular disease | 14 (32.6%) | NA | NA |
| BMI (kg/m^2^) | 24.8 ±3.9 | 24.3 ±3.8 | 25.7 ±4.0 |
| Fluid overload (L)* | 1.3 ±2.0 | 1.2 ±2.0 | 1.6 ±2.2 |
| SBP (mmHg) | 147.1 ±23.2 | 144.7 ±21.3 | 152.1 ±27.0 |
| DBP (mmHg) | 83.0 ±13.1 | 86.8 ±12.7 | 75.0 ±10.3 |
| RAAS inhibitor use | 18 (41.9%) | 13 (44.8%) | 5 (35.7%) |
| Statin use | 22 (51.2%) | 14 (48.3%) | 8 (57.1%) |
|  |  |  |  |
| *Advanced glycation endproducts and dicarbonyls* | | | |
| CML_free_ (nmol/L)* | 1142.2 [765.1-1538.3] | 1138.1 [769.3-1532.5] | 1177.9 [733.0-1538.9] |
| CML­_protein-bound_ (nmol/mmol lysine)* | 197.3 [163.1-256.6] | 187.3 [159.6-250.6] | 216.7 [179.1-324.5] |
| CEL_free_ (nmol/L)* | 734.7 [520.2-939.4] | 709.9 [496.0-907.9] | 816.8 [555.9-1163.5] |
| CEL_protein-bound_ (nmol/mmol lysine)* | 58.0 [41.8-74.3] | 58.1 [41.6-73.1] | 57.9 [42.2-83.2] |
| MG-H1_free_ (nmol/L)* | 2244.4 [1670.2-3154.6] | 2199.8 [1631.4-3176.9] | 2310.0 [2053.7-3203.8] |
| MG-H1­_protein-bound_ (nmol/mmol lysine)* | 59.1 [47.6-69.9] | 55.1 [46.4-68.9] | 63.6 [49.4-77.9] |
| GO (nmol/L)* | 1724.3 [1319.9-2157.4] | 1666.4 [2191.9-1889.8] | 2165.4 [1511.2-2583.4] |
| MGO (nmol/L)* | 1082.0 [856.6-1417.4] | 1003.4 [835.3-1251.3] | 1269.1 [1071.3-1533.8] |
| 3-DG (nmol/L)* | 1635.8 [1371.0-1948.5] | 1567.8 [1344.5-1821.1] | 1787.4 [1576.3-3085.4] |
| SAF (AU)* | 3.2 ±0.7 | 3.2 ±0.7 | 3.3 ±0.7 |
| *Serum biomarkers of endothelial dysfunction and low-grade inflammation* | | | |
| sVCAM-1 (μg/L) | 887.0 [690.0-1063.0] | 779.0 [655.5-934.0] | 948.5 [886.3-1225.5] |
| sE-selectin (μg/L) | 11.9 [7.8-16.3] | 12.3 [8.6-17.0] | 10.3 [6.9-15.1] |
| sP-selectin (μg/L) | 51.6 [37.2-63.0] | 55.0 [39.6-66.4] | 46.3 [32.2-57.4] |
| SThrombomodulin (μg/L) | 11.7 [9.5-13.8] | 11.0 [9.2-13.2] | 12.6 [10.4-14.3] |
| sICAM-1 (μg/L) | 415.0 [373.0-504.0] | 412.0 [362.0-477.5] | 473.5 [401.3-519.3] |
| sICAM-3 (μg/L) | 1.0 [0.8-1.3] | 1.0 [0.8-1.3] | 1.1 [0.8-1.3] |
| hs-CRP (mg/L) | 3.2 [1.2-7.6] | 2.6 [1.1-4.9] | 7.0 [1.8-12.6] |
| SAA (mg/L) | 6.8 [2.7-14.0] | 6.8 [3.2-14.3] | 7.6 [2.6-17.2] |
| IL-6 (ng/L) | 1.6 [0.7-2.6] | 1.1 [0.7-1.9] | 2.4 [1.5-4.2] |
| IL-8 (ng/L) | 13.8 [11.4-18.4] | 13.3 [10.8-17.4] | 14.8 [11.8-20.9] |
| TNF-α (ng/L) | 5.0 [4.2-6.0] | 4.7 [4.0-5.6] | 5.5 [4.5-6.2] |

Data are presented as n (%), mean ± standard deviation, or median [25^th^ percentile – 75^th^ percentile].

Abbreviations: 3-DG, 3-deoxyglucosone; AU, arbitrary units; BMI, body mass index; CEL, *N*^∈^(carboxyethyl)lysine; CML, *N*^∈^(carboxymethyl)lysine; DBP, diastolic blood pressure; eGFR_CKD-EPI_, estimated glomerular filtration rate based on the creatinine CKD-EPI equation; GO, glyoxal; HD, hemodialysis; hs-CRP, high-sensitivity C-reactive protein; IL-6, interleukin 6; IL-8, interleukin 8; KTx, kidney transplantation; MG-H1, *N*_δ_(5-hydro-5-methyl-4-imidazolon-2-yl)ornithine; MGO, methylglyoxal; NA, not applicable; PD, peritoneal dialysis; RAAS, renin angiotensin aldosterone system; SAA, serum amyloid A; SAF, skin autofluorescence; SBP, systolic blood pressure; sE-selectin, soluble E-selectin; sICAM-1, soluble intercellular adhesion molecule 1; sICAM-3, soluble intercellular adhesion molecule 3; sP-selectin, soluble P-selectin; sThrombomodulin, soluble Thrombomodulin; sVCAM-1, soluble vascular cell adhesion molecule 1; TNF-α, tumor necrosis factor alpha.

* Available in n = 42 for eGFR_CKD-EPI_, n = 38 for residual urine output (dichotomous), n = 27 for residual urine output (continuous), n = 42 for fluid overload, n = 42 for plasma AGEs, n = 38 for dicarbonyls, and n = 38 for skin autofluorescence.
