# Supplementary material for: Relations of advanced glycation endproducts and dicarbonyls with endothelial dysfunction and low-grade inflammation in individuals with end-stage renal disease in the transition to renal replacement therapy: A cross-sectional observational study
Source: PLoS One. 2019 Aug 13;14(8):e0221058. doi: 10.1371/journal.pone.0221058 (PMC6692010; doi:10.1371/journal.pone.0221058)
Supplement: S6 Table — (DOCX) [file pone.0221058.s008.docx]

S6 Table. Associations of advanced glycation endproducts and dicarbonyls with history of cardiovascular disease

|  |  | Cardiovascular disease | |
| --- | --- | --- | --- |
| Biomarker | Model | Odds ratio (95%CI) | *P* value |
| CML_free_ | 1 | 1.19 (0.61; 2.32) | 0.620 |
|  | 2 | 1.47 (0.62; 3.48) | 0.382 |
| CML­­_protein-bound_ | 1 | 1.66 (0.83; 3.29) | 0.149 |
|  | 2 | 1.42 (0.62; 3.26) | 0.408 |
| CEL­­_free_ | 1 | 1.66 (0.80; 3.42) | 0.173 |
|  | 2 | 2.38 (0.77; 7.41) | 0.133 |
| CEL_protein-bound_ | 1 | 0.98 (0.50; 1.89) | 0.943 |
|  | 2 | 1.16 (0.52; 2.62) | 0.715 |
| MG-H1_free_ | 1 | 1.20 (0.61; 2.35) | 0.595 |
|  | 2 | 1.35 (0.61; 2.99) | 0.463 |
| MG-H1_protein-bound_ | 1 | 1.54 (0.76; 3.10) | 0.227 |
|  | 2 | 1.55 (0.68; 3.54) | 0.299 |
| GO | 1 | 2.10 (0.89; 4.95) | 0.090 |
|  | 2 | 3.13 (0.97; 10.11) | 0.087 |
| MGO | 1 | 2.05 (0.91; 4.59) | 0.082 |
|  | 2 | 7.55 (1.26; 45.15) | 0.027 |
| 3-DG | 1 | 2.57 (0.99; 6.71) | 0.054 |
|  | 2 | 2.41 (0.77; 7.47) | 0.129 |
| SAF | 1 | 1.14 (0.58; 2.25) | 0.706 |
|  | 2 | 0.58 (0.23; 1.45) | 0.246 |

Odds ratios of history of cardiovascular disease are expressed per 1 standard deviation higher transformed levels of serum advanced glycation endproducts and serum dicarbonyls, and per 1 standard deviation higher skin autofluorescence. All serum advanced glycation endproducts and serum dicarbonyls were natural log transformed, except for free *N*^∈^(carboxymethyl)lysine (square root transformation), protein-bound MG-H1 (inverse transformation) and 3-deoxyglucosone (inverse transformation). In addition, skin autofluorescence was analyzed on its original scale.

Model 1: unadjusted analyses, model 2: adjusted for age and sex (diabetes mellitus was excluded due to the low number of affected participants).

Abbreviations: 3-DG, 3-deoxyglucosone; CEL, *N*^∈^(carboxyethyl)lysine; CML, *N*^∈^(carboxymethyl)lysine; GO, glyoxal; MG-H1, *N*_δ_(5-hydro-5-methyl-4-imidazolon-2-yl)ornithine; MGO, methylglyoxal; NA, not applicable; SAF, skin autofluorescence.

* Analyses based on n = 42 for serum AGEs, n = 38 for serum dicarbonyls and n = 38 for skin autofluorescence.
