# Supplementary material for: Relations of advanced glycation endproducts and dicarbonyls with endothelial dysfunction and low-grade inflammation in individuals with end-stage renal disease in the transition to renal replacement therapy: A cross-sectional observational study
Source: PLoS One. 2019 Aug 13;14(8):e0221058. doi: 10.1371/journal.pone.0221058 (PMC6692010; doi:10.1371/journal.pone.0221058)
Supplement: S7 Table — (DOCX) [file pone.0221058.s009.docx]

S7 Table. Associations of serum biomarkers of endothelial dysfunction and low-grade inflammation with history of cardiovascular disease

|  |  | Cardiovascular disease | |
| --- | --- | --- | --- |
| Biomarker | Model | Odds ratio (95%CI) | *P* value |
| sVCAM-1 | 1 | 2.30 (1.10; 4.84) | 0.027 |
|  | 2 | 1.68 (0.69; 4.06) | 0.253 |
| sE-selectin | 1 | 0.59 (0.30; 1.16) | 0.125 |
|  | 2 | 0.68 (0.30; 1.56) | 0.368 |
| sP-selectin | 1 | 0.69 (0.36; 1.34) | 0.278 |
|  | 2 | 0.69 (0.28; 1.68) | 0.414 |
| sThrombomodulin | 1 | 1.41 (0.72; 2.76) | 0.320 |
|  | 2 | 1.59 (0.65; 3.89) | 0.310 |
| sICAM-1 | 1 | 1.50 (0.77; 2.90) | 0.234 |
|  | 2 | 0.96 (0.43; 2.15) | 0.920 |
| sICAM-3 | 1 | 1.01 (0.53; 1.93) | 0.974 |
|  | 2 | 0.80 (0.36; 1.78) | 0.591 |
| hs-CRP | 1 | 1.61 (0.83; 3.13) | 0.158 |
|  | 2 | 1.13 (0.51; 2.47) | 0.767 |
| SAA | 1 | 0.99 (0.52; 1.89) | 0.974 |
|  | 2 | 0.66 (0.29; 1.52) | 0.332 |
| IL-6 | 1 | 1.93 (0.96; 3.87) | 0.065 |
|  | 2 | 1.85 (0.70; 4.89) | 0.212 |
| IL-8 | 1 | 1.22 (0.64; 2.35) | 0.547 |
|  | 2 | 1.06 (0.48; 2.35) | 0.883 |
| TNF-α | 1 | 1.56 (0.79; 3.09) | 0.199 |
|  | 2 | 2.03 (0.74; 5.51) | 0.167 |

Odds ratios of history of cardiovascular disease are expressed per 1 standard deviation higher transformed levels of serum biomarkers of endothelial dysfunction and low-grade inflammation. All serum biomarkers of endothelial dysfunction and low-grade inflammation were natural log transformed, except for interleukin 8 and tumor necrosis factor alpha (both inverse transformation).

Model 1: unadjusted analyses, model 2: adjusted for age and sex (diabetes mellitus was excluded due to the low number of affected participants).

Abbreviations: hs-CRP, high-sensitivity C-reactive protein; IL-6, interleukin 6; IL-8, interleukin 8; SAA, serum amyloid A; sE-selectin, soluble E-selectin; sICAM-1, soluble intercellular adhesion molecule 1; sICAM-3, soluble intercellular adhesion molecule 3; sP-selectin, soluble P-selectin; sThrombomodulin, soluble Thrombomodulin; sVCAM-1, soluble vascular cell adhesion molecule 1; TNF-α, tumor necrosis factor alpha.

* Analyses based on n = 43 for all serum biomarkers of endothelial dysfunction and low-grade inflammation.
