# Supplementary material for: Relations of advanced glycation endproducts and dicarbonyls with endothelial dysfunction and low-grade inflammation in individuals with end-stage renal disease in the transition to renal replacement therapy: A cross-sectional observational study
Source: PLoS One. 2019 Aug 13;14(8):e0221058. doi: 10.1371/journal.pone.0221058 (PMC6692010; doi:10.1371/journal.pone.0221058)
Supplement: S2 Fig — (DOCX) [file pone.0221058.s011.docx]

S2 Fig


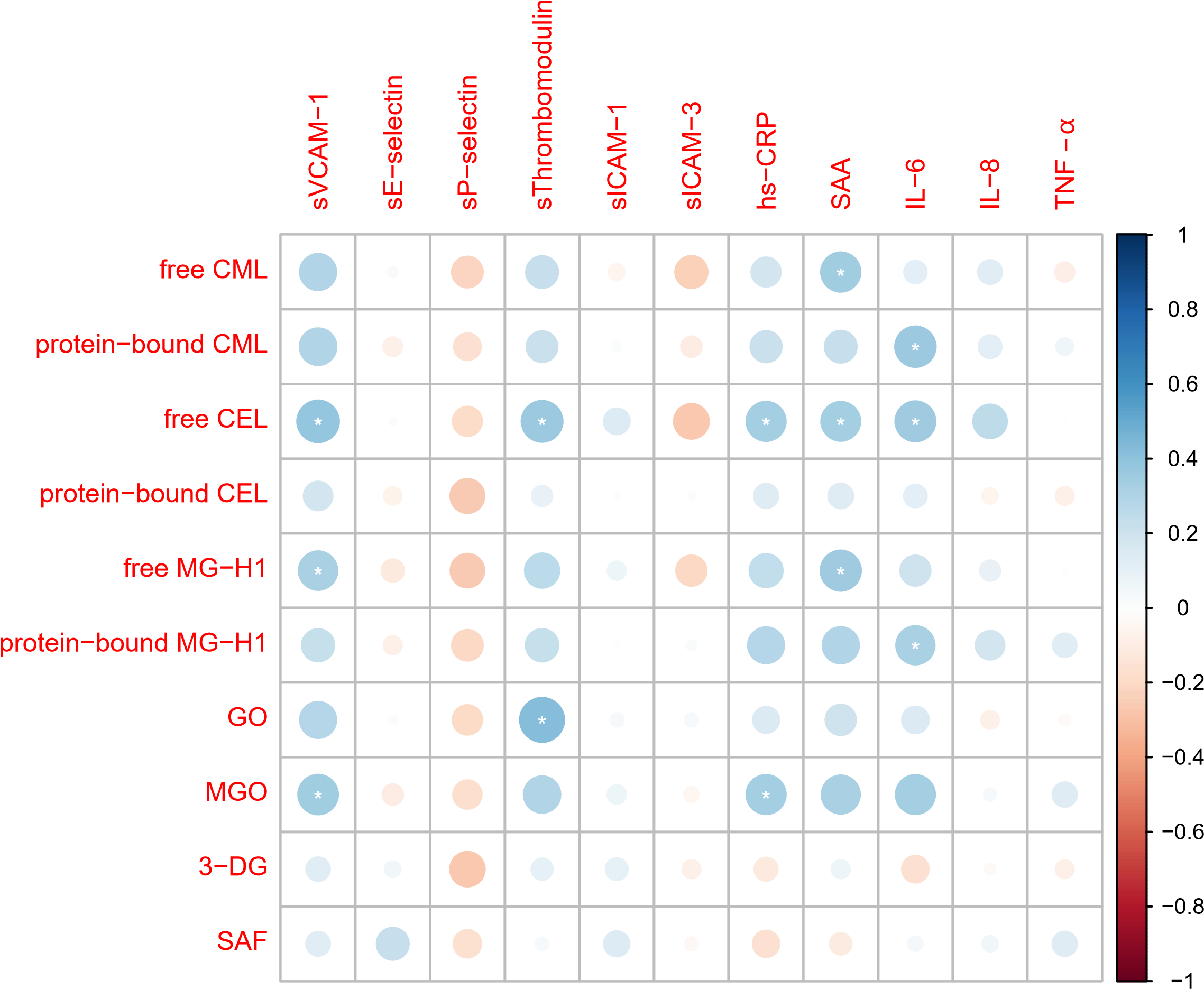


S2 Fig. Partial Spearman’s rank correlations of advanced glycation endproducts and dicarbonyls with individual serum biomarkers of endothelial dysfunction and low-grade inflammation after additional adjustment for fluid overload. Correlations are adjusted for age, sex, diabetes mellitus and fluid overload. Circle area and color indicate strength of Spearman’s rank correlation coefficients. Analyses are based on n = 42 for serum advanced glycation endproducts, n = 38 for serum dicarbonyls and n = 37 for skin autofluorescence. For abbreviations see main text. * *P* < 0.050.
