# Supplementary material for: Relations of advanced glycation endproducts and dicarbonyls with endothelial dysfunction and low-grade inflammation in individuals with end-stage renal disease in the transition to renal replacement therapy: A cross-sectional observational study
Source: PLoS One. 2019 Aug 13;14(8):e0221058. doi: 10.1371/journal.pone.0221058 (PMC6692010; doi:10.1371/journal.pone.0221058)
Supplement: S3 Fig — (DOCX) [file pone.0221058.s012.docx]

S3 Fig


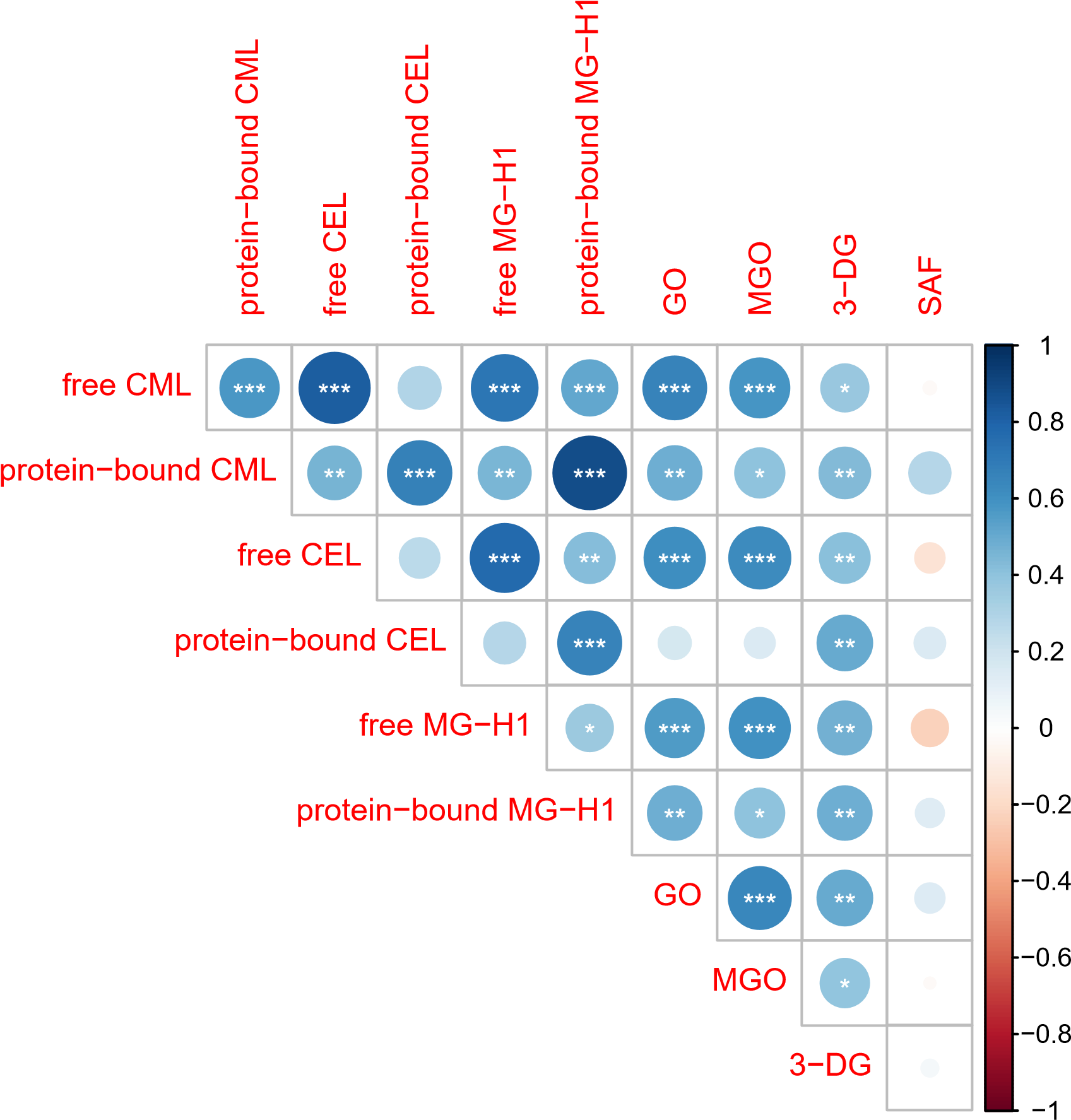


S3 Fig. Partial Spearman’s rank correlations among serum advanced glycation endproducts, serum dicarbonyls and skin autofluorescence. Correlations are adjusted for age, sex, and diabetes mellitus. Circle area and color indicate strength of Spearman’s rank correlation coefficients. Analyses are based on n = 33 (complete cases on the variables in the figure). For abbreviations see main text.

* *P* < 0.050; ** *P* < 0.010; *** *P* < 0.001.
